# Functional Differentiation and Regulatory Mechanisms of Ferrochelatases HemH1 and HemH2 in *Bacillus thuringiensis* Under Iron and Oxidative Stress

**DOI:** 10.3390/ijms26072911

**Published:** 2025-03-23

**Authors:** Jianghan Wang, Yi Luo, Tian Jiao, Shizhen Liu, Ting Liang, Huiting Mei, Shuang Cheng, Qian Yang, Jin He, Jianmei Su

**Affiliations:** 1 Hubei Key Laboratory of Regional Development and Environmental Response, Faculty of Resources and Environmental Science, Hubei University, Wuhan 430062, China; 202321108012145@stu.hubu.edu.cn (J.W.); luoyi031622@163.com (Y.L.); 202421108012135@stu.hubu.edu.cn (S.L.); 202231108031008@stu.hubu.edu.cn (T.L.); 202221108012247@stu.hubu.edu.cn (H.M.); 202221108012295@stu.hubu.edu.cn (S.C.); 202131108031001@stu.hubu.edu.cn (Q.Y.); 2 National Key Laboratory of Agricultural Microbiology, College of Life Science and Technology, Huazhong Agricultural University, Wuhan 430062, China; 2024400382@buct.edu.cn (T.J.); hejin@mail.hzau.edu.cn (J.H.)

**Keywords:** ferrochelatase, heme synthesis, physiological function, oxidative stress, metabolic regulation

## Abstract

Ferrochelatase is the terminal enzyme in heme biosynthesis. *Bacillus thuringiensis* (Bt) 97-27 contains two ferrochelatases, HemH1 and HemH2, but their regulatory mechanisms and functional differences under virous environmental stimuli remain unclear. This study confirmed that the iron uptake regulator protein (Fur) bound to the promoters of *hemH1* and *hemH2*, with Fe^2+^ or Fe^3+^ enhancing this binding. Heterologous expression of HemH1 and HemH2 in *Escherichia coli* showed that pEH2/BL grew better than pEH1/BL under different 2,2′-Bipyridyl, Fe^2+^, and Fe^3+^ concentrations. Under iron limitation, the heme precursor ALA production decreased significantly in both strains. The heme production of pEH2/BL decreased sharply under iron-limited conditions, while that of pEH1/BL decreased significantly under iron-rich conditions. The H_2_O_2_ sensitivity experiment revealed that *E. coli* pEH1/BL was more tolerant to H_2_O_2_ than pEH2/BL. In Bt, Δ*hemH2* was most sensitive to H_2_O_2_ stress, but complementation of *hemH1* or *hemH2* partially restored H_2_O_2_ resistance, with the overexpressed strain pHH2/Bt being most tolerant. β-galactosidase assays indicated that Fur positively regulated *hemH1* and negatively regulated *hemH2* under normal conditions, but this regulation reversed with 2.5 mM Fe^3+^. qRT-PCR showed upregulation of genes related to heme synthesis, oxidative stress, and ferrous iron transport. This study reveals the functional differentiation of HemH1 and HemH2 under the joint regulation of Fur and environmental factors, highlighting their synergistic roles in heme synthesis, heavy metal detoxification, and oxidative stress resistance to maintain bacterial physiological homeostasis.

## 1. Introduction

Heme, an iron-containing porphyrin macrocycle, is also known as hematin or iron porphyrin and belongs to the family of cyclic modified tetrapyrroles [1]. As a prosthetic group for numerous metalloenzymes [2], heme plays critical roles in cellular signaling, cell differentiation, gene transcription regulation, and protein translation [3,4,5,6]. Due to its high bioavailability, no side effects, and easy of absorption, heme is widely utilized as a food additive and a natural iron supplement in the food industry, medicine, and biotechnology fields [7,8,9]. Currently, three distinct heme biosynthesis pathways have been identified in prokaryotes: the protoporphyrin-dependent (PPD) pathway, the coproporphyrin-dependent (CPD) pathway, and the siroheme-dependent (SHD) pathway [10]. The PPD pathway is predominantly found in eukaryotes and most Gram-negative bacteria, while the CPD pathway, is primarily present in Gram-positive bacteria. The SHD pathway (initially termed the alternative heme biosynthesis pathway, Ahb) is active in sulfate-reducing *Proteobacteria* and *Archaea* [11].

Ferrochelatase, encoded by the *hemH* gene, is the terminal enzyme in heme biosynthesis. In the PPD pathway, protoporphyrin ferrochelatase catalyzes the insertion of Fe^2+^ into protoporphyrin IX ring to form heme. In the CPD pathway, coproporphyrin ferrochelatase (CpfC) incorporates Fe^2+^ into coproporphyrin III to generate Fe-coproheme, which is subsequently decarboxylated to form heme [12]. Notably, the SHD pathway does not require ferrochelatase for heme synthesis. At present, most bacteria contain only one HemH. The existence of two ferrochelatase paralogues, HemH1 and HemH2, has been reported only in *Shewanella* species [13,14]. For instance, in *S. loihica* PV-4, disrupting *hemH1*, rather than *hemH2*, leads to a significant accumulation of the heme precursor, extracellular protoporphyrin IX (PPIX). Notably, *hemH2* compensates for the loss of *hemH1* by driving heme and cytochrome synthesis in the Δ*hemH1* strain, albeit with reduced intracellular heme levels [13]. However, *S. oneidensis* MR-1 exhibits distinct regulatory mechanisms. The deletion of *hemH1* or *hemH2* alone does not cause PPIX accumulation. PPIX accumulates only in the Δ*hemH1*Δ*hemH2* double mutant under aerobic conditions. Furthermore, *hemH1* exhibits constitutive expression, and its disruption triggers significant transcriptional upregulation of *hemH2* to compensate for functional deficits [13,14]. The transcription of the heme synthesis gene *hemH1* is regulated by the housekeeping sigma factor RpoD and potentially by OxyR, while that of *hemH2* appears to be regulated by the oxidative stress-associated sigma factor RpoE2 [14]. These findings suggest that while the two ferrochelatases share functional redundancy in heme biosynthesis with compensatory interactions, they are subjected to differential regulatory mechanisms under environmental stresses to maintain heme homeostasis.

The central ferrous ion coordinated within heme is crucial for its electron transfer capacity and redox activity. Studies have shown that iron levels can influence heme synthesis to some extent [15,16]. The ferric uptake regulator (Fur) protein plays a pivotal role in iron metabolism regulation [17]. As a DNA-binding protein, Fur recognizes specific DNA sequences and utilizes Fe^2+^ or Mn^2+^ as a corepressor to modulate the transcription of target genes [18]. Under iron-deficient conditions, Fur cannot bind Fe^2+^ and exists in its apo-Fur form, allowing RNA polymerase to bind to promoter regions and initiate gene transcription [19]. Conversely, under iron-sufficient conditions, the Fur binds to Fe^2+^ to form a complex and binds to promoter regions, preventing RNA polymerase binding and thereby repressing the transcription of target genes such as *endd*, *entF*, *entCEBA*, and *iucABCD* [20]. Previous studies have reported that overexpression of the *furA* gene in *Anabaena* sp. activates the transcription and translation of the ferrochelatase gene *hemH*, indicating a regulatory role of FurA in heme biosynthesis [19]. However, the specific mechanisms by which Fur regulates *hemH* in other bacterial strains, as well as the influence of metal ions, remain poorly understood. Additionally, whether the *hemH* gene participates in other physiological functions beyond its role as a ferrochelatase is still unclear.

*Bacillus thuringiensis* serovar *konkukian* strain 97-27 (hereafter, Bt 97-27), ubiquitously distributed in soil, aquatic systems, and insect intestinal tracts, also has two ferrochelatases, HemH1 and HemH2 like *Shewanella*. These characteristics make Bt 97-27 an ideal system for studying heme synthesis and homeostasis in environmental microbes. However, the molecular mechanisms underlying functional divergence between HemH1 and HemH2 in Bt 97-27 remain elusive, and their differential transcriptional regulatory networks under varying physiological conditions are yet to be elucidated. To address these knowledge gaps, we first constructed *hemH1* and *hemH2* heterologous expression strains, designated as pEH1/BL and pEH2/BL, along with an empty vector control strain (pE/BL) in *Escherichia coli* BL21(DE3). Subsequently, we generated a *hemH2* knockout strain (Δ*hemH2*), an empty vector control strain (pH/Bt), an overexpression strain (pHH2/Bt), and complemented strains (pHH1/Δ*h2* and pHH2/Δ*h2*) in Bt 97-27. Systematic functional analyses were performed to dissect the roles of HemH1 and HemH2 in antioxidant stress response, heavy metal detoxification, and heme synthesis. Furthermore, the regulatory mechanisms of the Fur protein on *hemH1* and *hemH2* under the action of various environmental stressors, including 2,2′-bipyridyl (Dip), Fe^2+^, Fe^3+^, and H_2_O_2_, were also investigated. This study not only advances our understanding of the complexity of heme biosynthesis regulatory networks in bacterial environmental adaptation but also provides potential mechanistic insights into human pathologies such as anemia, porphyria, and cancer.

## 2. Results

### 2.1. The Effect of Different Concentrations of Dip, Fe^2+^, and Fe^3+^ on Strain Growth

**Figure 1:**
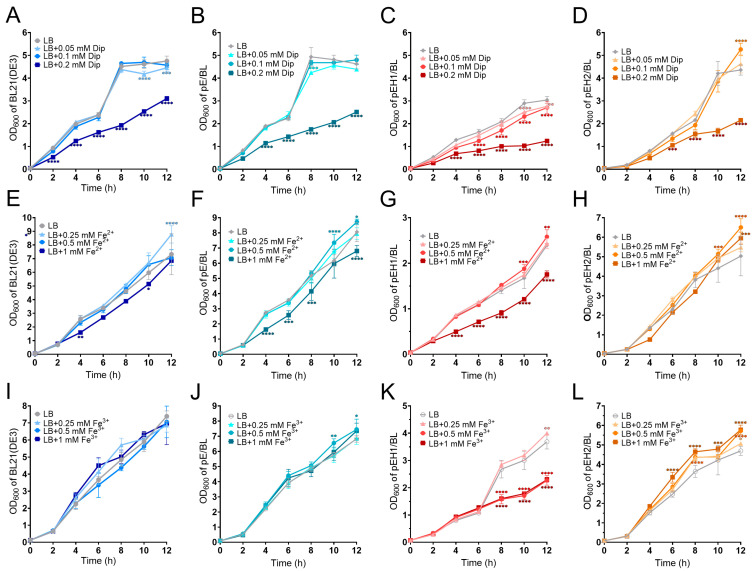
Growth curves of four *E. coli* strains under varying concentrations of Dip, Fe^2+^, and Fe^3+^. (**A**–**D**) Growth curves of BL21(DE3), pE/BL, pEH1/BL, and pEH2/BL under different concentrations of Dip. (**E**–**H**) Growth curves of BL21(DE3), pE/BL, pEH1/BL, and pEH2/BL under different concentrations of Fe^2+^. (**I**–**L**) Growth curves of BL21(DE3), pE/BL, pEH1/BL, and pEH2/BL under different concentrations of Fe^3+^. Among them, *p* < 0.05 indicates a statistical difference. “*” indicates *p* < 0.05; “**” indicates *p* < 0.01; “***” indicates *p* < 0.001; “****” indicates *p* < 0.0001.

The growth curve analysis of different *E. coli* strains showed that, during the initial 8 h, the host strain BL21(DE3), the empty vector strain pE/BL, and the recombinant strains pEH1/BL and pEH2/BL exhibited nearly identical growth trends (Figure S1A). However, from 10 to 168 h, the growth of the recombinant strain pEH1/BL was significantly inhibited, while the growth of the recombinant strain pEH2/BL was significantly promoted compared to other strains (Figure S1B). When the Dip concentration was ≤0.1 mM, it had a minimal impact on the growth of the host strain BL21(DE3) (Figure 1A), the empty vector strain pE/BL (Figure 1B), and the recombinant strain pEH2/BL (Figure 1D) at the vast majority of times. However, the growth of the recombinant strain pEH1/BL was moderately inhibited, and this inhibitory effect intensified with increasing Dip concentrations (Figure 1C). When the Dip concentration reached 0.2 mM, the growth of all four strains was significantly suppressed (Figure 1A–D).

The growth curves of the four *E. coli* strains under different Fe^2+^ concentrations are shown in Figure 1E–H. When the Fe^2+^ concentration was ≤0.5 mM, it moderately promoted the growth of all four strains, which was especially significant at 12 h (Figure 1E–H). However, when the Fe^2+^ concentration reached 1 mM, it significantly inhibited the growth of the empty vector strain pE/BL (Figure 1F) and the recombinant strain pEH1/BL (Figure 1G), but had little effect on the growth of the host strain BL21 (DE3) (Figure 1E) and the recombinant strain pEH2/BL (Figure 1H), with only significant effects at certain time points (Figure 1H).

The growth curves of the four *E. coli* strains under different Fe^3+^ concentrations are shown in Figure 1I–L. For the host strain BL21(DE3) and the empty vector strain pE/BL, varying Fe^3+^ concentrations did not exhibit significant effects on their growth within 8 h (Figure 1I,J). For the recombinant strain pEH1/BL, Fe^3+^ concentrations ≥0.5 mM significantly inhibited its growth after 8 h (Figure 1K). In contrast, for the recombinant strain pEH2/BL, Fe^3+^ concentrations ranging from 0.25 to 1 mM promoted its growth, and the promoting effect increased with higher Fe^3+^ concentrations (Figure 1L).

**Figure 2:**
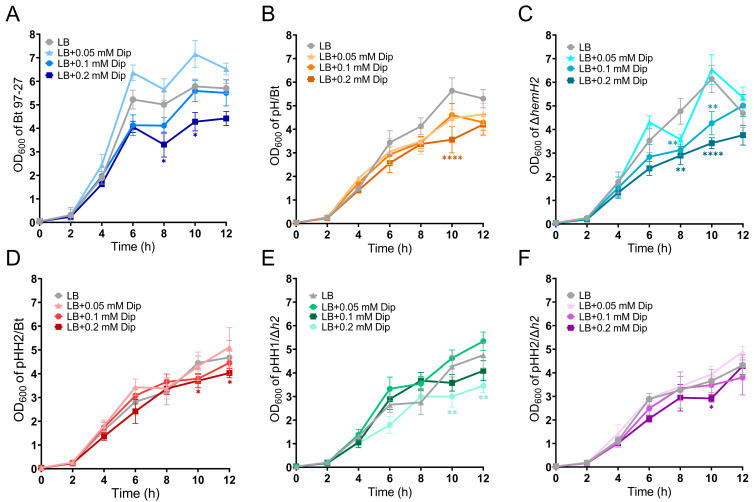
Growth curves of six *Bacillus thuringiensis* (Bt) strains under varying concentrations of Dip. (**A**) Wild-type strain Bt 97-27. (**B**) Empty vector strain pH/Bt. (**C**) Knockout strain Δ*hemH2*. (**D**) Overexpression strain pHH2/Bt. (**E**) Complemented strain pHH1/Δ*h2*. (**F**) Complemented strain pHH2/Δ*h2*. Among them, *p* < 0.05 indicates a statistical difference. “*” indicates *p* < 0.05; “**” indicates *p* < 0.01; “****” indicates *p* < 0.0001.

In Bt strains, the addition of Fe^2+^ and Fe^3+^ at concentrations ranging from 0 to 1 mM had minimal impact on bacterial growth. Therefore, this study focused on examining the effects of iron-limited conditions induced by varying concentrations of Dip on the growth of different strains (Figure 2A–F). The results demonstrated that, compared to other strains, the overexpression strain pHH2/Bt and the complemented strains pHH1/Δ*h2* and pHH2/Δ*h2* all grew slower in LB containing erythromycin due to the presence of pHT315 plasmid (Figure 2D–F). When Dip concentrations were ≤0.05 mM, there was no significant effect on the growth of all the strains. When the Dip concentration was 0.1 mM, only the growth of the knockout strain Δ*hemH2* was significantly inhibited. When the Dip concentration was 2mM, all strains showed significant inhibitory effects from the eighth hour onwards at different time points (Figure 2A–F).

### 2.2. The Effect of Different Concentrations of H_2_O_2_ on Bacterial Growth

**Figure 3:**
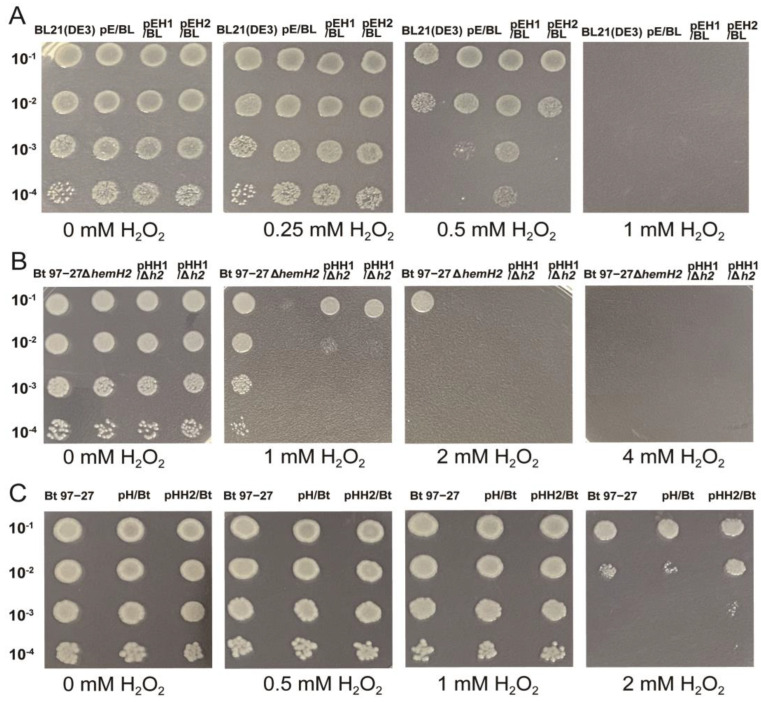
Sensitivity experiments of four *E. coli* and six Bt strains to different concentrations of H_2_O_2_. (**A**) *E. coli* strains BL21(DE3), pE/BL, pEH1/BL, and pEH2/BL grown on LB plates containing different concentrations of H_2_O_2_. (**B**) Wild-type strain Bt 97-27, knockout strain Δ*hemH2*, complemented strain pHH1/Δ*h2*, and complemented strain pHH2/Δ*h2* grown on LB plates containing different concentrations of H_2_O_2_. (**C**) Wild-type strain Bt 97-27, empty vector strain pH/Bt and overexpression strain pHH2/Bt grown on LB plates containing different concentrations of H_2_O_2_.

The H_2_O_2_ sensitivity experiment found that on LB solid plates containing 0.25 mM H_2_O_2_, the four dilution gradients of *E. coli* BL21(DE3), pE/BL, pEH1/BL, and pEH2/BL strains all grew normally (Figure 3A). On LB solid plates containing 0.5 mM H_2_O_2_, the host strain BL21(DE3) and the recombinant strain pEH2/BL with dilutions exceeding 10^−3^ could not grow, while the empty vector strain pE/BL and the recombinant strain pEH1/BL could grow, with pEH1/BL growing even better. Therefore, the recombinant strain pEH2/BL is more sensitive to H_2_O_2_ than the empty vector strain pE/BL and the recombinant strain pEH1/BL. However, on LB solid plates containing 1 mM H_2_O_2_, none of the four strains could grow (Figure 3A).

This study also examined the sensitivity of six Bt strains to H_2_O_2_. It was found that on solid plates containing 1 mM H_2_O_2_, at a dilution of 10^−2^, only Δ*hemH2* could not grow, although pHH1/Δ*h2* and pHH2/Δ*h2* also showed poor growth. At a dilution of 10^−3^, only Bt 97-27 could grow, while the other three strains could not, indicating that the H_2_O_2_ tolerance of the knockout strain Δ*hemH2* was significantly reduced compared to the wild-type strain Bt 97-27. However, when the complemented recombinant plasmids pHT315-*hemH1* and pHT315-*hemH2* were introduced into Δ*hemH2*, the ability of complemented strains pHH1/Δ*h2* and pHH2/Δ*h2* to resist H_2_O_2_ was significantly enhanced, although they still could not be restored to the level of the wild-type strain Bt 97-27 (Figure 3B). On LB solid plates containing 2 mM H_2_O_2_, Bt 97-27, pH/Bt, and pHH2/Bt could all grow, with pHH2/Bt growing the best, but Δ*hemH2*, pHH1/Δ*h2*, and pHH2/Δ*h2* could not grow (Figure 3B,C). Therefore, compared to the wild-type strain Bt 97-27 and the empty vector strain pH/Bt, the overexpressed strain pHH2/Bt is the most tolerant to H_2_O_2_, while Δ*hemH2* is the most sensitive to H_2_O_2_.

### 2.3. The Effect of Iron-Limited Conditions on the Production of 5-ALA by E. coli Strains

**Figure 4:**
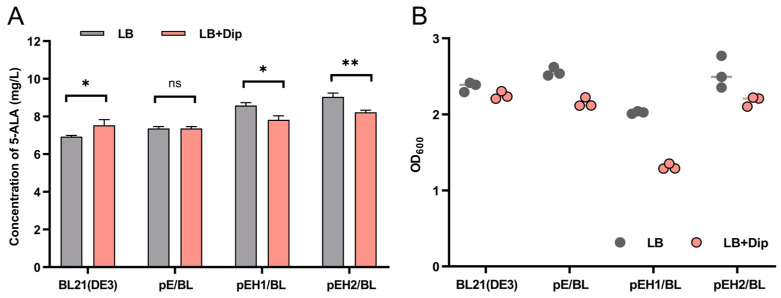
5-ALA yield and OD_600_ values of four *E. coli* strains under LB and iron-limited LB conditions. (**A**) 5-ALA yield of the four strains cultivated in LB with 0 mM and 0.2 mM Dip. (**B**) OD_600_ of the four strains when sampling for 5-ALA measurement. Among them, “ns” indicates *p* > 0.05, no statistical difference; *p* < 0.05 indicates a statistical difference. “*” indicates *p* < 0.05; “**” indicates *p* < 0.01.

Under iron-limited conditions created by adding 0.2 mM Dip to LB medium, the ALA production of the host strain BL21(DE3) (7.53 mg/L) was significantly higher than that without Dip (6.94 mg/L) (*p* < 0.05). In contrast, the ALA production of the empty vector strain pE/BL (7.37 mg/L) showed no significant change compared to that without Dip (7.37 mg/L). However, the ALA production of the recombinant strains pEH1/BL (7.83 mg/L) and pEH2/BL (8.22 mg/L) significantly decreased compared to their respective productions without Dip (8.59 mg/L and 9.05 mg/L, *p* < 0.05) (Figure 4A). Although the addition of Dip led to a relative decrease in the cell density of pEH1/BL, it had no significant effect on the cell density of pEH2/BL. This indicates that the reduction in ALA production is not directly correlated with changes in cell density (Figure 4B).

### 2.4. The Effect of Dip, Fe^2+^, Fe^3+^ on the Production of Heme by E. coli Strains

**Figure 5:**
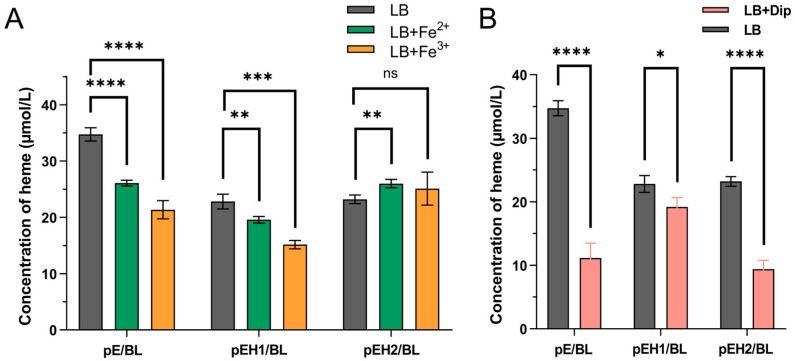
Heme production of three *E. coli* strains under different cultivation conditions. (**A**) Bacterial heme productions in LB and LB containing 0.1 mM Fe^2+^ and 0.025 mM Fe^3+^, respectively. (**B**) Bacterial heme productions in LB and LB containing 0.2 mM Dip. Among them, “ns” indicates *p* > 0.05, no statistical difference; *p* < 0.05 indicates a statistical difference. “*” indicates *p* < 0.05; “**” indicates *p* < 0.01; “***” indicates *p* < 0.001; “****” indicates *p* < 0.0001.

As shown in Figure 5A, the empty vector strain pE/BL can produce 34.74 µM heme in LB medium, while the heme content decreases to 26.08 µM and 21.35 µM in LB containing 0.1 mM Fe^2+^ and 0.025 mM Fe^3+^, respectively. The recombinant strain pEH1/BL can produce 22.82 µM heme in LB, while the heme production decreases to 19.58 µM and 15.16 µM in LB containing 0.1 mM Fe^2+^ and 0.025 mM Fe^3+^, respectively. The recombinant strain pEH2/BL can produce 23.21 µM heme in LB. In LB containing 0.1 mM Fe^2+^ and 0.025 mM Fe^3+^, the heme production increases to 25.99 µM and 25.12 µM, respectively, maintaining an upward or stable trend.

According to Figure 5B, the empty vector strain pE/BL can produce 37.47 µM heme in LB, but its yield decreases to 11.17 µM under iron-limiting conditions with 0.2 mM Dip. The recombinant strain pEH2/BL can produce 23.20 µM heme in LB, but its yield sharply decreases to 9.39 µM/L under iron-limited conditions with 0.2 mM Dip. The recombinant strain pEH1/BL can produce 22.82 µM heme in LB, but its heme yield decreases to 19.19 µM under iron-limited conditions with 0.2 mM Dip.

### 2.5. Metal Ion-Dependent Fur Binding to P_hemH1_ or P_hemH2_ Promoters and Binding Region Verification

The SDS-PAGE results demonstrated that the recombinant Fur protein purified by Ni-NTA (19.3 mg/mL) exhibited high purity and matched the theoretical molecular weight of Fur (21.68 kDa) (Figure S2), which was suitable for subsequent electrophoretic mobility shift assay (EMSA) experiments.

**Figure 6:**
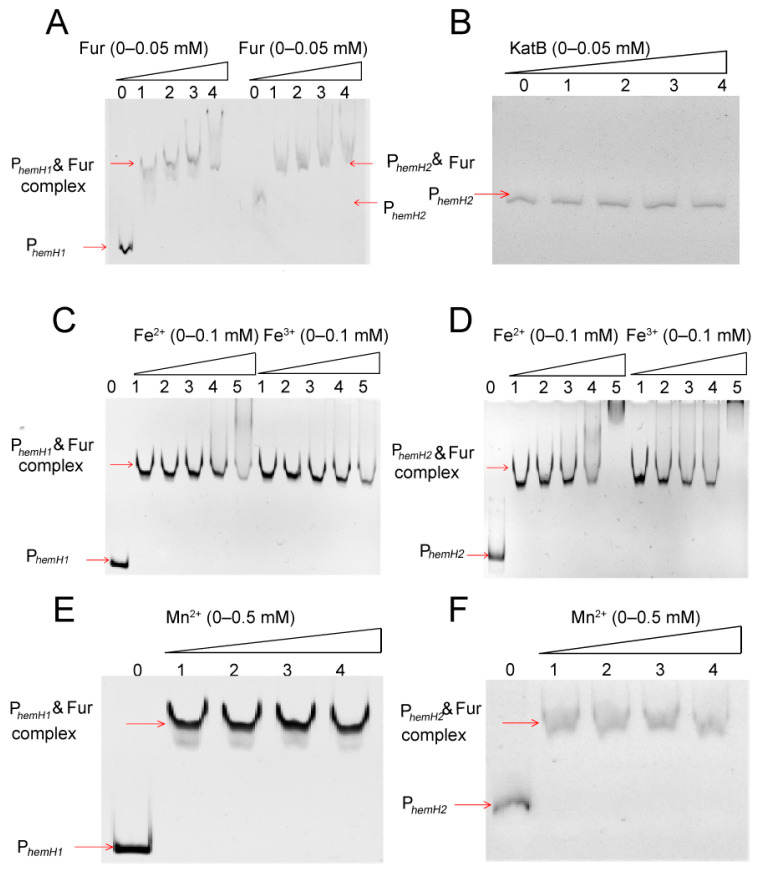
EMSA experiment. (**A**,**B**) Various concentrations of Fur or KatB binding with P*_hemH1_* or P*_hemH2_*. (**C**,**D**) Effects of varying concentrations of Fe^2+^ or Fe^3+^ on the binding of Fur to P*_hemH1_* or P*_hemH2_*. (**E**,**F**) Effects of varying concentrations of Mn^2+^ on the binding of Fur to P*_hemH1_* or P*_hemH2_*.

As shown in Figure 6A, for both P*_hemH1_* and P*_hemH2_*, the migration of the promoter DNA bands slowed down and shifted upward with increasing concentrations of the Fur protein. This indicates that Fur, as a regulatory protein, can bind to the promoter DNA of P*_hemH1_* and P*_hemH2_*, forming Fur–P*_hemH1_* and Fur–P*_hemH2_* complexes, respectively. Moreover, higher concentrations of Fur enhanced the binding affinity between Fur and the promoter DNA. In contrast, the non-regulatory protein KatB (catalase) did not bind to P*_hemH2_*, as no band shift was observed even with increasing concentrations of KatB (Figure 6B).

Figure 6C,D displayed that as the concentrations of Fe^2+^ or Fe^3+^ in the EMSA reaction system increased, the binding between Fur and the promoter DNA became stronger, while the amount of free promoter DNA fragments decreased. This suggests that both Fe^2+^ and Fe^3+^ promoted the binding of Fur to P*_hemH1_* and P*_hemH2_*, and this promoting effect intensified with increasing concentrations of Fe^2+^ or Fe^3+^. However, as shown in Figure 6E,F, increasing concentrations of Mn^2+^ had no effect on the binding of Fur to either P*_hemH1_* or P*_hemH2_*.

### 2.6. Verification of the Binding Regions of Fur with P_hemH1_ and P_hemH2_ with Fur by DNase I Footprinting

**Figure 7:**
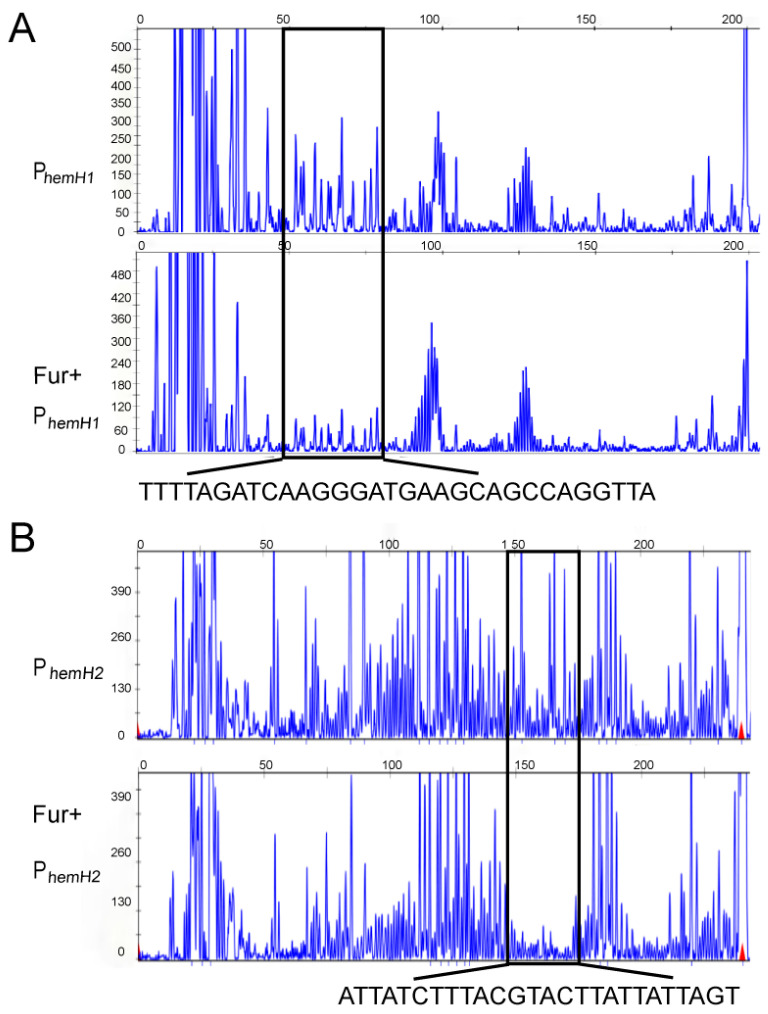
DNase I footprinting analysis. (**A**) DNase I footprinting sequence analysis before and after Fur protein binding to the P*_hemH1_* promoter DNA. (**B**) DNase I footprinting sequence analysis before and after Fur protein binding to the P*_hemH2_* promoter DNA. The regions marked with black boxes indicate the areas protected by the Fur protein, with the corresponding DNA sequences labeled below.

The DNase I footprinting assay showed that the binding region between Fur and the P*_hemH1_* promoter DNA was located at 50–80 bp of the P*_hemH1_* promoter DNA fragment (Figure 7A), with the specific sequence being TTTTAGATCAAGGGATGAAGCAGCCAGGTTA. The confirmed binding sequence between Fur and the P*_hemH1_* promoter partially overlaps with the previously predicted Fur box (GGTTATATCTT) in the P*_hemH1_* promoter fragment (the overlap is indicated by the underline), located right at the front of the predicted Fur box. The binding region of P*_hemH2_* promoter DNA with Fur was located at the 150–175 bp of the P*_hemH2_* promoter (Figure 7B), with the specific sequence being ATTATCTTTACGTACTTATTATTAGT. The confirmed binding sequence between Fur and the P*_hemH2_* promoter overlaps partially with the previously predicted Fur box (AGAATAATTATC) in the P*_hemH2_* promoter fragment (the overlap is indicated by the underline), located just after the predicted Fur box.

### 2.7. The Regulation Mode of Fur on hemH1 and hemH2 and Its Influence by Fe^3+^

**Figure 8:**
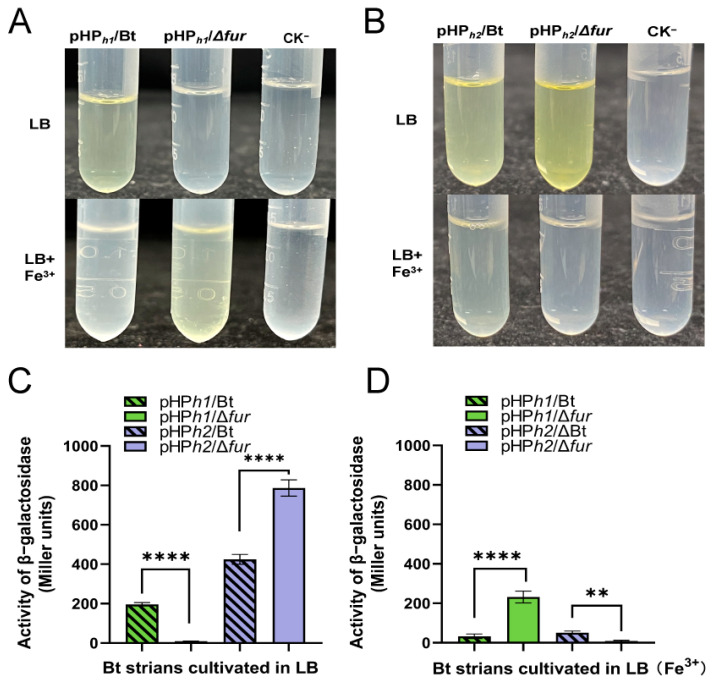
Effects of 2.5 mM Fe^3+^ on the β-galactosidase activity of four Bt reporter strains. (**A**) Images of β-galactosidase activity of pHP*_h1_*/Bt and pHP*_h1_*/Δ*fur* strains in LB culture with or without Fe^3+^. (**B**) Images of β-galactosidase activity of pHP*_h2_*/Bt or pHP*_h2_*/Δ*fur* strains in LB culture with or without Fe^3+^. (**C**) Detection of β-galactosidase activity of four Bt strains in LB culture. (**D**) Detection of β-galactosidase activity of four Bt strains in LB culture with Fe^3+^. Among them, “ns” indicates *p* > 0.05, no statistical difference; *p* < 0.05 indicates a statistical difference. “**” indicates *p* < 0.01; “****” indicates *p* < 0.0001.

The β-galactosidase activity assay revealed that in LB medium, the activity of β-galactosidase in pHP*_h1_*/Bt was higher than that in pHP*_h1_*/Δ*fur*, indicating that Fur in pHP*_h1_*/Bt could bind to P*_hemH1_*, thereby activating the expression of the downstream reporter gene *lacZ*. However, in LB medium supplemented with 2.5 mM Fe^3+^, the β-galactosidase activity of pHP*_h1_*/Bt (44.81 M.U.) was significantly lower than that of pHP*_h1_*/Δ*fur* (275.72 M.U.) (Figure 8A). Furthermore, in LB medium containing 2.5 mM Fe^2+^, no β-galactosidase activity was detected in either pHP*_h1_*/Bt or pHP*_h1_*/Δ*fur* (Figure S3). These results suggest that in the presence of Fe^3+^, Fur in pHP*_h1_*/Bt binds to P*_hemH1_*, partially repressing or downregulating the expression of the downstream gene *lacZ*. In contrast, in the presence of Fe^2+^, the expression of the downstream reporter gene is completely repressed.

The pHP*_h2_*/Bt and pHP*_h2_*/Δ*fur* strains were cultured in LB medium supplemented with 2.5 mM Fe^2+^ or 2.5 mM Fe^3+^ for 8 h to measure β-galactosidase activity. At the 21 min mark of the reaction, the samples of pHP*_h2_*/Bt and pHP*_h2_*/Δ*fur* without Fe^3+^ turned yellow. The β-galactosidase activity of pHP*_h2_*/Bt was 438.12 M.U., while that of pHP*_h2_*/Δ*fur* was 786.73 M.U. After more than 2 h of reaction, a faint yellow color was observed in the pHP*_h2_*/Bt sample supplemented with Fe^3+^, with a β-galactosidase activity of 70.86 M.U. (Figure 8B). Additionally, in LB medium containing 2.5 mM Fe^2+^, no β-galactosidase activity was detected in either pHP*_h2_*/Bt or pHP*_h2_*/Δ*fur* (Figure S3). These experimental results demonstrate that in LB medium, the activity of P*_hemH2_* in Bt 97-27 is lower than that in Δ*fur*. In LB medium supplemented with Fe^3+^, the activity of P*_hemH2_* in Bt 97-27 is higher than that in Δ*fur*. However, in LB medium containing Fe^2+^, no activity of P*_hemH2_* is detected in both Bt 97-27 and Δ*fur*.

### 2.8. qRT-PCR Results

**Figure 9:**
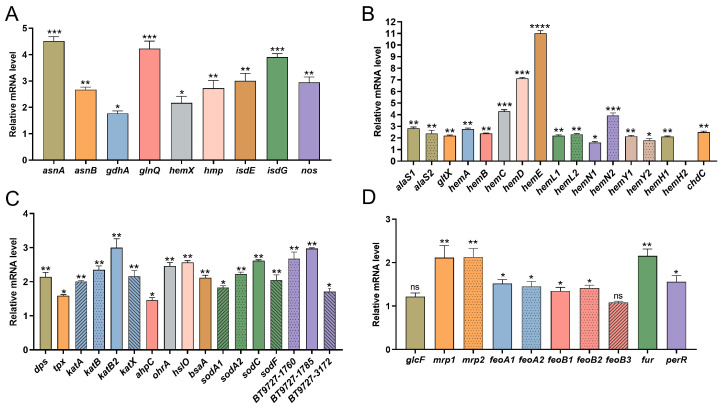
Relative expression levels of genes in the Δ*hemH2* strain compared with Bt 97-27. (**A**) Relative expression levels of heme-related genes. (**B**) Relative expression levels of genes in the fecoporphyrin heme synthesis pathway. (**C**) Relative expression levels of oxidative stress-related genes. (**D**) Relative expression levels of ferrous transport-related genes. Among them, *p* < 0.05 indicates a statistical difference. “*” indicates *p* < 0.05; “**” indicates *p* < 0.01; “***” indicates *p* < 0.001; “****” indicates *p* < 0.0001.

As shown in Figure 9, the fold changes in gene expression were normalized relative to a reference gene, with 16S rRNA used as the internal control and Bt 97-27 as the comparator. The qRT-PCR results revealed that, compared with Bt 97-27, the relative mRNA levels of nine heme-related genes in the Δ*hemH2* strain were significantly upregulated, These included genes involved in heme biosynthesis and modification (*hemX*), heme uptake and catabolism (*isdE, isdG*), nitrogen metabolism in the heme synthesis pathway *(asnA*, *asnB*, *gdhA*, *glnQ*), heme detoxification and oxidative stress response (*hmp*), and heme-related signaling (*nos*). Notably, genes such as *asnA*, *glnQ*, and *isdG* exhibited particularly pronounced upregulation (Figure 9A).

Genes associated with the coproporphyrin-dependent heme synthesis pathway included those encoding ALA precursor synthesis (*hemA*, *hemL1*, *hemL2*, *alaS1*, *alaS2*, *gltX*), porphyrin backbone assembly (*hemB*, *hemC*, *hemD*), coproporphyrinogen modification (*hemE*, *hemN1*, *hemN2*), protoporphyrinogen oxidation (*hemY1*, *hemY2*), protoporphyrin IX iron chelation (*hemH1*, *hemH2*), and coproporphyrin branch regulation (*chdC*). Compared to Bt 97-27, the relative mRNA levels of 16 coproporphyrin-dependent heme synthesis pathway genes were significantly upregulated in the Δ*hemH2* strain, with the exception of *hemH2*, which was not expressed. The top four most highly expressed genes were *hemE*, *hemD*, *hemC*, and *hemN2* (Figure 9B).

In the Δ*hemH2* strain, the relative mRNA levels of 17 oxidative stress-related genes were all significantly upregulated compared to Bt 97-27. These included catalase genes (*katA*, *katB*, *katB2*, *katX*, *ahpC*), peroxidase genes (*tpx*, *bsA*), superoxide dismutase genes (*sodA1*, *sodA2*, *sodC*, *sodF*, *ohrA*), DNA-binding protein genes (*dps*), heat shock protein genes (*hslQ*), and nitroreductase genes (*BT9727-1760*, *BT9727-1785*, *BT9727-3172*). Notably, genes such as *katB2*, *BT9727-1785*, *BT9727-1760*, *sodC*, *hslO*, and *ohrA* showed particularly strong upregulation. These findings suggest that *hemH2* plays a critical role in antioxidant stress responses, and its deletion activates the relative expression of other oxidative stress-related genes (Figure 9C).

Regarding iron transport-related genes, including those encoding ferrous iron transporters (*feoA1*, *feoA2*, *feoB1*, *feoB2, feoB3*), iron–sulfur cluster carrier proteins (*mrp1*, *mrp2*), siderophore-related genes (*glcF*), and iron uptake regulatory proteins (*fur*, *perR*), the relative mRNA levels of all genes except *feoB3* and *glcF* were significantly upregulated in the Δ*hemH2* strain compared to Bt 97-27. Notably, genes encoding iron–sulfur cluster carrier proteins (*mrp1*, *mrp2*) and iron uptake regulatory proteins (*fur*) exhibited particularly strong upregulation (Figure 9D).

## 3. Discussion

### 3.1. The Sequence Analysis of Ferrochelatases from Different Strains

In Bt 97-27, we identified two ferrochelatases, HemH1 and HemH2, also known as CpfC1 and CpfC2. We first performed multiple-sequence alignment analyses of HemH1 and HemH2 with homologs from species containing one or two HemH enzymes (Figure S4). The selected species are either well characterized for HemH or represent typical model strains, including Gram-positive bacteria (*Staphylococcus aureus*, *Streptococcus* sp., *Bacillus subtilis*) [21,22,23] and Gram-negative bacteria (*Escherichia coli* BL21(DE3), *Pseudomonas aeruginosa*, *Shewanella* PV-4 and MR-1) [13,14,24,25]. These species have been extensively studied in the context of heme biosynthesis and iron metabolism, making them ideal comparators for sequence analysis. This study found a 63.5% sequence identity between HemH1 and HemH2 in Bt 97-27. HemH1 exhibited sequence similarities of 19.73%, 21.30%, 51.0%, 28.9%, 71.8%%, 21.3%, and 24.5% with the ferrochelatases of *Shewanella loihica* PV-4 (ABO23011), *Shewanella oneidensis* MR-1 (AAN55069.2), *Staphylococcus aureus* (ABD73483.1), *Streptococcus* (WP_023944239.1), *Bacillus subtilis* (SPY20679.1), *Pseudomonas* (WP_003171634.1), and *E. coli* BL21(DE3) (CAQ30948.1), respectively. Similarly, HemH2 showed sequence similarities of 21.1%, 22.2%, 46.3%, 30.0%, 58.9%, 16.2%, and 21.3% with the ferrochelatases of the same organisms, respectively. Generally, when sequence identity exceeds 65–80%, enzyme functions are highly conserved, with nearly identical catalytic mechanisms and substrate specificity. Although bacterial and mammalian ferrochelatases often exhibit low sequence identity (e.g., ~10% between humans and *B. subtilis*), their core regions retain similar secondary and tertiary structures [26]. Thus, HemH1 and HemH2 in Bt 97-27 likely retain the core ferrochelatase function shared across species, namely facilitating metal ion insertion into porphyrin to produce heme.

However, even among HemHs with divergent overall sequence identities, conservation of residues in catalytic active sites, substrate-binding pockets, or cofactor-binding regions may preserve the same substrate specificity and core catalytic mechanism, though catalytic efficiency or substrate preferences may vary [27]. Reports in the literature indicate that ferrochelatase catalytic rates (*k_cat_*) differ significantly across species, typically ranging from 0.11 to 15.3 min^−1^. Notably, *S. aureus* HemH exhibits a *k_cat_* of 165 min^−1^ when using coproporphyrin III and Fe^2+^ as substrates 28], while B. subtilis HemH shows conflicting *k_cat_* values of 78 min^−1^ [28] and 0.11 min^−1^ [29]. Furthermore, the catalytic function of B. subtilis HemH relies on cooperative interactions among residues such as Tyr13, His183, and Glu272. Mutations at these sites markedly alter metal specificity, catalytic efficiency, or substrate-binding capacity. For example, the Y13M mutation (tyrosine to methionine) shifts metal specificity, accelerating Co^2+^ insertion while abolishing Cu^2+^ utilization [22], and Glu272 mutation eliminates the Mg^2+^-mediated stimulation of Zn^2+^ insertion into deuteroporphyrin IX [23]. Therefore, the enzymatic function of HemH is not only influenced by sequence similarity, but also by other environmental factors such as co-factors, substrate concentration, enzyme stability and kinetic parameters.

### 3.2. The Effect of Different Concentrations of Dip, Fe^2+^, and Fe^3+^ on the Function of Ferrochelatases HemH1 and HemH2

Our research found that the functional differences between HemH1 and HemH2 are related to the levels of ferrous and ferric ions in the environment. The growth curve results of the recombinant *E. coli* strains indicated that the high expression of the ferrous chelator HemH2 promoted the growth of the recombinant strain pEH2/BL, while the high expression of HemH1 inhibited the growth of the recombinant strain pEH1/BL. However, under appropriate iron-limiting conditions (≤0.1 mM Dip), the growth of the recombinant strain pEH1/BL was somewhat inhibited, while the growth of the recombinant strain pEH2/BL was not affected, but under extremely iron-limiting conditions, the growth of both strains is affected. Similarly, under extreme iron restriction conditions, the growth of Bt 97-27, the empty vector strain pH/Bt, and the knockout strain Δ*hemH2* was also inhibited. This may have been because under iron-deficient conditions, the iron content within microorganisms decreases, leading to reduced enzyme activity necessary for the synthesis of DNA, RNA, and proteins, thereby limiting microbial growth and reproduction. Li also reported that in LB medium supplemented with Dip, the intracellular iron levels in bacterial strains were significantly reduced, resulting in a marked inhibition of growth [30,31]. Under low concentrations of Fe^2+^ (≤0.5 mM) or Fe^3+^ (≤0.25 mM), the growth of recombinant strains pEH1/BL and pEH2/BL was promoted to some extent, which is consistent with literature reports that low concentrations of iron could promote bacterial growth and reproduction [32]; however, supplementing with high concentrations of Fe^2+^ (1 mM) or Fe^3+^ (≥0.5 mM) had no effect or even had a promoting effect on the growth of recombinant strain pEH2/BL, but it inhibited the growth of recombinant strain pEH1/BL. This might be because the high concentration of iron ions triggered the Fenton reaction in pEH1/BL, causing toxicity to the strain [33]. Therefore, within an appropriate range, whether in iron-deficient or iron-rich (Fe^2+^ or Fe^3+^) environments, the expression of HemH2 significantly enhances the growth and tolerance of the recombinant strain pEH2/BL.

Ferrochelatase is a crucial enzyme in heme biosynthesis. However, in our heme synthesis experiments, the heterologous expression of both HemH1 and HemH2 led to a reduction in heme production in *E. coli.* Studies have shown that overexpression of the hemH gene in *E. coli* does not increase heme synthesis but instead suppresses it [29]. Similarly, Kwon et al. reported that heterologous expression of the *hemH* gene from *Bacillus subtilis* in *E. coli* failed to enhance heme production [34]. Additionally, Franken et al. observed no significant increase in heme synthesis when overexpressing the *hemH* gene in *Aspergillus niger* [35]. It is hypothesized that the *hemH* gene may be subject to post-transcriptional feedback regulation, resulting in reduced ferrochelatase activity and, consequently, an inability to effectively increase heme yield.

Iron, as a cofactor for ferrochelatase, is crucial for the enzyme’s activity and function. Consequently, iron limitation or excess can also affect the production of heme synthesis precursors, such as ALA, and heme itself in recombinant strains. Under iron-limited conditions, the ALA production in the host strain BL21(DE3) increased, while it decreased in the recombinant strains pEH1/BL and pEH2/BL. This observation aligned with findings by Li et al., who reported a decrease in ALA production under Dip iron-limited conditions after overexpressing ferrochelatase in *E. coli* [31]. The reduction in ALA precursors in pEH1/BL and pEH2/BL might be attributed to the expression of ferrochelatases HemH1 and HemH2 under iron-limited conditions, which promoted the conversion of ALA to downstream heme, thereby preventing the cytotoxic accumulation of ALA and ensuring normal heme synthesis in *E. coli*. Under iron-limited conditions, heme production in pEH2/BL decreased sharply, while the decline in pEH1/BL was more gradual. In contrast, in the presence of 0.1 mM Fe^2+^ or 0.025 mM Fe^3+^, heme production in pEH1/BL decreased, whereas it remained stable or increased in pEH2/BL. Hobbs et al. found that adding 0.3–0.7 μL of Fe^2+^ significantly increased the Km value of ferrochelatase in *Staphylococcus aureus*, suggesting that elevated iron concentrations enhance the Km value of coproporphyrin III [27]. Similarly, Li et al., reported that the addition of 0.1 mM FeSO_4_ significantly reduced the accumulation of ALA while increasing heme accumulation, which aligns with our findings [36]. Furthermore, Wang et al. demonstrated that exogenous addition of 100 μmol/L Fe^2+^ effectively converted PPIX into heme, increasing the heme content to 29.44 μmol/g-DCW, compared to conditions without exogenous Fe^2+^. Similarly, the addition of 25 μmol/L Fe^3+^ also significantly enhanced heme production, reaching a concentration of 38.22 μmol/g-DCW [37]. These results indicate that under iron-limited conditions, ferrochelatase HemH1 plays a critical role in heme synthesis, whereas HemH2 becomes more important under iron-replete conditions.

Knocking out *hemH2* in Bt 97-27 upregulated the expression of genes involved in heme synthesis, particularly *hemE*, *hemD*, and *hemC*, suggesting that the knockout of *hemH2* most significantly affected the process from porphobilinogen to coproporphyrinogen III in the heme biosynthesis pathway. Additionally, the genes encoding asparagine synthetase (*asnA*) and glutamine transporter (*glnQ*), both critical for ALA synthesis, were significantly upregulated. This indicates that *hemH2* knockout also impacts the production of the heme precursor ALA. In *Riemerella anatipestifer*, knockout of the *hemH* gene leads to a deficiency in heme required for respiratory processes, significantly impairing respiratory function. This heme deficiency not only affects metabolic activities and the tricarboxylic acid cycle but also upregulates the expression of genes encoding heme-binding proteins [38].

### 3.3. The Role of hemH1 and hemH2 in Antioxidant Stress

The H_2_O_2_ sensitivity experiment found that among the *E. coli* strains, the recombinant strain pEH1/BL was the most tolerant to H_2_O_2_, followed by the empty vector strain pE/BL, the recombinant strain pEH2/BL, and the host strain BL21(DE3). This indicates that the expression of ferrochelatase HemH1 can significantly enhance the ability of the recombinant strain pEH1/BL to resist H_2_O_2_, whereas the expression of HemH2 does not improve the resistance of the recombinant strain pEH2/BL to H_2_O_2_. In Bt, compared with the wild-type strain Bt 97-27 and the empty vector strain pH/Bt, the overexpressed strain pHH2/Bt was the most tolerant to H_2_O_2_, while Δ*hemH2* was the most sensitive to H_2_O_2_, indicating that HemH2 played an important role in the oxidative stress response of *Bt*. Through sequence analysis, we found that the downstream gene of the *hemH2* gene in the Bt 97-27 strain was the catalase *katB* gene. Catalase can decompose H_2_O_2_ into water and oxygen, protecting cells from the toxic effects of H_2_O_2_ [39]. To verify whether the oxidative stress capacity of the *hemH2* gene was related to the function of the *katB* gene, we designed primers *katB-1151580*, *hemH-1150700*, and *hemH-1151000* within the *katB* gene and the *hemH2* gene. The PCR amplification and sequencing results indicate that the *hemH2* gene and the downstream *katB* gene are co-transcribed in the Bt 97-27 strain (Figure S5A,B). Therefore, knocking out the *hemH2* gene may affect the transcription of the downstream *katB* gene. The transcription of the gene therefore makes the Δ*hemH2* knockout strain most sensitive to H_2_O_2_ stress. In fact, after knocking out the *katB* gene, we indeed found that the Δ*katB* strain was less tolerant to H_2_O_2_ than the Δ*hemH2* strain (Figure S5C).

After complementing the *hemH1* or *hemH2* gene in Δ*hemH2*, both pHH1/Δ*h2* and pHH2/Δ*h2* could partially restore the ability to resist H_2_O_2_, indicating that HemH1 and HemH2 played a protective role in the strain’s defense against H_2_O_2_ damage, and there is a compensatory mechanism between the two in resisting oxidative stress damage. Dai found that there were also two ferrochelatases in *Shewanella* sp. PV-4, with the encoded gene *hemH1* playing a more crucial role in the oxidative stress response, while *hemH2* might serve as a backup to maintain heme dynamic balance [13]. Under normal growth conditions, *hemH1* is a dominant gene, and when it is absent, *hemH2* can serve as a substitute [14].

This compensatory phenomenon was confirmed by qRT-PCR results. After knocking out *hemH2*, in addition to the upregulation of *hemH1* gene expression, the expression of other antioxidant system related genes was also upregulated to compensate for the loss of the antioxidant stress function of the *hemH2* gene, thereby reducing oxidative damage to the strain. Among the four catalase genes detected, *katA*, *katB*, *katB2*, and *katX*, the relative expression level of *katB2* was the highest, rather than the *katB* gene downstream of *hemH2*. This indicates that the expression of the *katB* gene is not entirely influenced by the upstream *hemH2* gene; it has its own independent promoter and can still participate in antioxidant activity even after *hemH2* knockout. Superoxide dismutase (SOD) is an antioxidant enzyme that contains metal ions (such as Cu/Zn, Mn, Fe). Its main function is to catalyze the conversion of superoxide anions (O^2−^) into oxygen and more stable H_2_O_2_ [40,41]. This study found that among the four genes, *sodA1*, *sodA2*, *sodF*, and *sodC*, the relative expression level of *sodC* was the highest. Nitro reductase belongs to the flavoprotein family and can directly or indirectly eliminate ROS, protecting cells from oxidative damage, or it can indirectly affect the activity of antioxidant enzymes (such as SOD and catalase) by regulating the ratio of NAD(P)H/NAD(P)^+^ [42]. This study found that among the nitro-reductase family protein genes *BT9727_1760*, *BT9727_1785*, and *BT9727_3172*, the relative expression levels of *BT9727_1760* and *BT9727_1785* were both relatively high.

### 3.4. Differential Regulatory Mechanisms of Fur on hemH1 and hemH2

Fur is a global transcriptional regulator present in the vast majority of bacteria which uses Fe^2+^ as a cofactor to maintain iron homeostasis within bacteria by regulating iron uptake and storage systems. Studies have confirmed that Fur can participate in the Fe^2+^ transport and the heme synthesis pathway [43,44], but there are very few reports on the regulation of *hemH1* and *hemH2* by Fur. This study first verified through EMSA experiments that Fur could indeed bind to the promoters of *hemH1* and *hemH2*, and the increase of Fe^2+^ and Fe^3+^ concentrations promoted the binding of Fur to P*_hemH1_* and P*_hemH2_*. Next, through DNase I footprinting experiments, the identified binding sequences were basically aligned with the predicted Fur boxes positions, but the sequences of Fur boxes were not the same. Although the Fur box is usually considered one of the key criteria for determining whether a gene is regulated by Fur, in bacteria, the DNA sequences actually recognized by Fur exhibit a certain degree of conservation and varying levels of diversity [45,46]. For example, a study in 2014 on *E. coli* demonstrated that Fur bound to the different DNA sequences when activating or repressing target genes [47]. Additionally, in the case of *Griffithsia* sp., the crystal structure of the complex formed by Fur binding to the DNA of the feo AB1 promoter region (an atypical Fur box sequence) has also been discovered [48]. These findings indicate that the DNA sequences actually bound by Fur are more diverse and complex than the known Fur boxes box is more diverse and complex.

**Figure 10:**
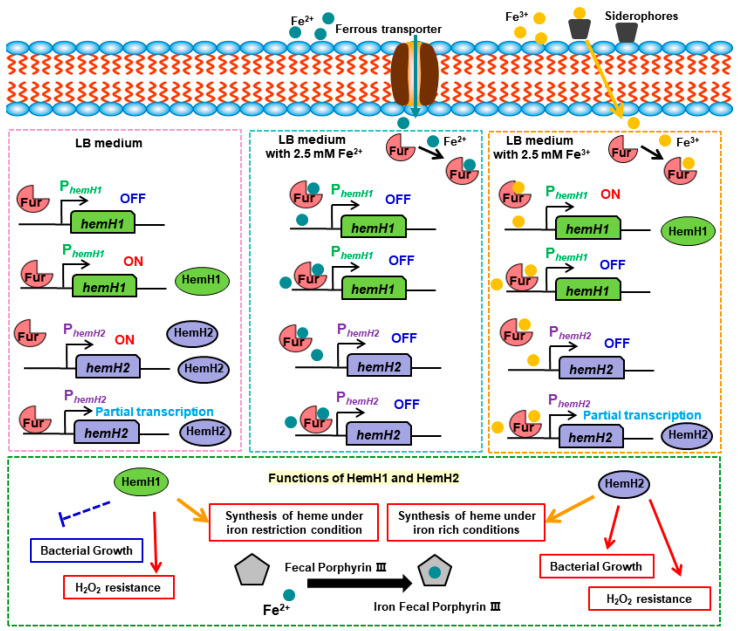
The regulatory mechanisms of Fur on *hemH1* and *hemH2*, and the functions of HemH1 and HemH2.

When Fur binds to the DNA of target genes, it can inhibit or activate the expression of certain genes. In this study, β-galactosidase activity assays revealed that under normal conditions (LB), Fur exhibited a positive regulatory mode on *hemH1* and a negative regulatory mode on *hemH2*. However, in the presence of 2.5 mM Fe^3+^, Fur exhibited a negative regulatory mode on *hemH1* and a positive regulatory mode on *hemH2* (Figure 10). Studies have shown that in the presence of sufficient iron content, the apo-Fur protein undergoes structural changes, transforming into the holo-Fur dimer form, which tightly binds to the promoter region of the target gene. This binding creates spatial hindrance that inhibits RNA polymerase, thereby suppressing the transcriptional activity of iron uptake-related genes. In an iron-deficient environment, Fur protein cannot effectively bind to the target gene region, leading to the restoration of the previously suppressed transcriptional activity of iron uptake genes, further enhancing the bacteria’s efficiency in iron absorption [48,49]. However, in the presence of 2.5 mM Fe^2+^, the β-galactosidase activity of these Bt reporter strains was not detected in this study, indicating that the *hemH1* and *hemH2* genes could not be expressed in environments rich in Fe^2+^ (Figure S3). It is speculated that the high iron ion concentration repression mechanism of holo-Fur may have been activated, meaning that when the iron ion concentration inside the bacteria is too high or reaches equilibrium, Fur will bind with Fe^2+^, acting as an inhibitor and attaching to the promoter region of the target gene. This binding hinders RNA polymerase from binding to the target gene, thereby inhibiting the expression of the target gene [16].

Although our study has elucidated the functional divergence between HemH1 and HemH2, numerous details remain to be explored. For instance, the precise molecular mechanism underlying the iron-mediated differential regulation of HemH1 and HemH2 remains unclear. Furthermore, the regulatory networks governing *hemH1* and *hemH2* expression under varying iron conditions warrant systematic investigation through integrated transcriptomic and proteomic analyses. Such comprehensive approaches would provide valuable insights into the complex regulatory mechanisms controlling porphyrin biosynthesis homeostasis.

## 4. Materials and Methods

### 4.1. Strains, Plasmids, and Culture Conditions

*E. coli* and *Bacillus thuringiensis* (Bt 97-27) were cultured in Luria–Bertani medium [50]. *E. coli* was cultured at 37 °C and 200 rpm, while *Bacillus thuringiensis* was cultured at 28 °C and 200 rpm. The bacterial strains and plasmids used in this study are listed in Table S1.

### 4.2. Construction of E. coli Recombinant Strains Expressing fur and hemH

Using Bt 97-27 genomic DNA as a template, the *fur* gene was amplified by PCR with the primer pair *fur*-F (*Bam*H I) and *fur*-R (*Xho* I); the *hemH1* gene was amplified with the primer pair *hemH1*-F (*Nco* I) and *hemH1*-R (*Xho* I); and the *hemH2* gene was amplified with the primer pair *hemH2*-F (*Nco* I) and *hemH2*-R (*Xho* I). The oligonucleotide primers used in this study are listed in Table S2. The above PCR products were digested with corresponding restriction endonucleases (Takara, Beijing, China), and then ligated with T4 DNA Ligase (Thermo Scientific, Waltham, MA, USA) to the linearized pET28b plasmid. The connected product was then transformed into *E. coli* BL21(DE3) to obtain the pEF/BL recombinant strains of *fur* (Figure S6), pEH1/BL recombinant strains of *hemH*1 (Figure S7), and pEH2/BL recombinant strains of *hemH*1 (Figure S8). After PCR validation and sequencing, we transferred the *E. coli* recombinant strains to LB medium containing 100 mg/mL Kanamycin (Kan) and cultured it at 37 °C at 200 rpm, respectively. When the OD_600_ of the bacterial cells reached 0.6–0.8, 0.25 mM IPTG was added and cultivated at 28 °C for 12–16 h to induce the expression of the recombinant proteins. The Ni-NTA method was used to purify the recombinant protein Fur [51,52].

### 4.3. Construction of hemH Knockout, Overexpression, and Complementation Strains

The markerless gene deletion system was successfully developed for Bt 97-27 based on an I-*Sce*I-mediated replacement method as established in *B. anthracis* by Janes and Stibitz (2006) [53]. The detailed procedures have been well described in a previous publication [52,54]. We used primers *hemH2*-UF (*Kpn* I), *hemH2*-UR, *hemH2*-DF and *hemH2*-DR (*Mlu* I) (see Table S2) to amplify the sequences of the upstream and downstream arms of the *hemH2* gene; these were amplified separately. Next, the overlapping extension PCR technique was used to synthesize the upstream and downstream sequences of the *hemH2* gene, namely the UD sequence. Then, the UD fragment was digested with the corresponding restriction endonucleases, and then ligated into the linearized pRP1028 plasmid using T4 DNA Ligase to construct the *hemH2* gene knockout plasmid pRP1028-*hemH2*-UD. This plasmid was then transformed into *E. coli* DH5α to obtain the pRH2/DH strain (see Figure S9). Using a triparental mating system containing pRH2/DH (donor strain), DH5α containing the helper plasmid pSS1827 (helper strain) and Bt 97-27 (recipient strain) was used to form the pRH2/DH plasmid-integrated Bt 97-27 strain by homologous single cross-over recombination. The I-*Sce* I endonuclease resulted in a chromosomal double-stranded break and thus stimulated host genetic repair by homologous recombination between the flanking repeat sequences [44,55]. Positive deletion strains of *hemH2* were verified by PCR and sequencing, which obtained the *hemH2* gene knockout strain (∆*hemH2*). Deletion of *fur* was performed using the same method.

We used primers OE*hemH2*-F (*EcoR* I) and OE*hemH2*-R (*Hind* III) for PCR to amplify the *hemH2* gene (Table S2). Then, double digestion of the pHT315 plasmid and *hemH2* gene fragment with *EcoR* I and *Hind* III, followed by ligation with T4 DNA Ligase, occurred. The constructed pHT315-*hemH2* overexpression plasmid was then electroporated into Bt 97-27 to obtain the *hemH2* overexpression strain (pHH2/Bt) (Figure S10A,B). Using the same method, the recombinant plasmid pHT315-*hemH1* was also constructed (Figure S10C). The electroporator (Bio-Rad, Gene Pulser Xcell™) parameters were as follows: voltage, 2300 V; capacitance, 25 μF; resistance, 200 Ω; cuvette, 1 mm.

The correctly sequenced recombinant plasmids pHT315-*hemH1* and pHT315-*hemH2* were electroporated into ∆*hemH2* competent cells, resulting in two complemented strains of the ∆*hemH2* strain, pHH1/∆*h2* and pHH2/∆*h2*. All the aforementioned Bt strains were cultured in LB at 28 °C, with pHH2/Bt, pHH1/Δ*h2*, and pHH2/Δ*h2* strains requiring the addition of erythromycin (Ery) at a final concentration of 25 μg/mL.

### 4.4. Determination of the Growth Curve of the Strain

This study explored the effects of different iron-rich and iron-limited conditions on the growth of the *E. coli* host strain BL21(DE3), the empty vector strain pE/BL, and the recombinant strains pEH1/BL and pEH2/BL. We set the Dip concentrations to 0, 0.05, 0.1, and 0.2 mM; set the Fe^2+^ concentrations to 0, 0.25, 0.5, and 1 mM; and set the Fe^3+^ concentrations to 0, 0.25, 0.5, and 1 mM. After overnight activation of the above strains at 37 °C, we transferred them to LB medium at a concentration of 1% of inoculum (adding 50 μg/mL Kan, if necessary). The recombinant strains also required the addition of 0.2 mM IPTG when the OD_600_ was approximately 0.6–0.8 (about 2 h). We recorded the initial OD_600_ value as 0 h, took samples every 2 h to measure the OD_600_, and continuously monitored them for 12 h. All growth curve assays were performed with triplicate technical replicates for each treatment group, which underwent batch-to-batch validation.

At the same time, the effects of different iron-limiting conditions on Bt 97-27, the knockout strain Δ*hemH2*, the empty vector strain pH/Bt, the overexpression strain pHH2/Bt, and the complemented strains pHH1/Δ*h2* and pHH2/Δ*h2* were also explored. The aforementioned strains were transferred to 100 mL of LB medium (adding 25 μg/mL Ery, if necessary) at an initial OD_600_ of 0.01, followed by the addition of different concentrations of Dip, and cultivated at 28 °C. For the first 24 h, the OD_600_ was measured every 2 h, and then measured every 24 h for seven days.

### 4.5. H_2_O_2_ Tolerance Experiment

After transferring the activated *E. coli* BL21(DE3), empty strain pE/BL, and recombinant vector strains pEH1/BL and pEH2/BL in a certain proportion to LB medium (adding 50 μg/mL Kan, if necessary), they were cultured at 37 °C, 200 rpm until OD_600_ = 0.6. After diluting it in a 10^−1^ to 10^−4^ gradient, 3 μL of the above bacterial solution was taken and spotted onto solid LB plates containing 0, 0.25, 0.5, and 1 mM H_2_O_2_ respectively, The growth of the strains was observed after culturing at 37 °C for 12 h.

We transferred the activated Bt 97-27, knockout strain Δ*hemH2*, empty vector strain pH/Bt, overexpression strain pHH2/Bt, complemented strain pHH1/Δ*h2*, and pHH2/Δ*h2* in a certain ratio, and cultured them at 28 °C, 200 rpm until OD_600_ = 0.6. After diluting in a 10^−1^ to 10^−4^ gradient, we took 3 μL of the above bacterial solution and spotted it on solid LB plates containing different concentrations of H_2_O_2_ (0–4 mM). We observed the growth of the strains after culturing them at 28 °C for 12 h.

### 4.6. Determination of Heme and 5-Aminolevulinic Acid Concentrations

The activated *E. coli* BL21(DE3), empty vector strain pE/BL, and recombinant strains pEH1/BL and pEH2/BL were individually transferred in specific proportions to LB medium containing 0.1 mM Fe^2+^, 0.025 mM Fe^3+^, and 0.2 mM Dip, and cultured at 37 °C, 200 r/min. Among them, when the recombinant strains pEH1/BL and pEH2/BL were cultured to OD_600_ ≈ 0.6 (approximately 2 h), 0.2 mM IPTG was added and induced for 5 h. We took a certain amount of each bacterial sample, centrifuged it at 4 °C, 8000× *g* for 1 min, and then discarded the supernatant. We added 500 μL of 20 mM oxalic acid solution to suspend the cells, and let them stand in the dark at 4 °C for 16 h. Then, we added 500 μL of 2 M oxalic acid solution and mixed it thoroughly. We took half of the sample and heated it at 95 °C for 30 min to fully react hemin with oxalic acid and convert it into porphyrin; we placed the other half of the sample reacting at room temperature as a control group. Then, we centrifuged it at 6000× *g* for 5 min, took 200 μL of the supernatant and added it to a black 96-well plate to measure the fluorescence value (Arbitrary Units), with the excitation wavelength set at 400 nm and the emission wavelength set at 620 nm. We calculated the heme content according to the standard curve of heme and fluorescence values [56].

We transferred the above four strains of *E. coli* to LB medium (adding 50 μg/mL Kan, if necessary) containing 0.2 mM Dip. Then, we investigated the content of 5-aminolevulinic acid (ALA), the heme synthesis precursor, under iron-limited conditions for each strain. We took an appropriate amount of each bacterial culture, and centrifuged it at 4 °C, 8000× *g* for 1 min. We added 500 μL of 2 M acetate buffer (pH 4.6) and 250 μL of 99% acetylacetone to the supernatant. We mixed this well and heated it at 100 °C for 15 min. After cooling, we added 1 mL of Ehrlich’s reagent (Solabio, China), reacted it for 20 min, and measured the ALA content at OD_554_ according to the ALA standard curve [57].

### 4.7. Gel Mobility Shift Assay (EMSA) and DNase I Footprinting Assay

Using Bt 97-27 genomic DNA as a template, primers P*_hemH1_*-F and P*_hemH1_*-R, and primers P*_hemH2_*-F and P*_hemH2_*-R were used to PCR amplify approximately 200 bp DNA fragments upstream of the *hemH1* and *hemH2* genes, which were used as the P*_hemH1_* and P*_hemH2_* promoter regions, respectively. The purified P*_hemH1_* and P*_hemH2_* promoter DNA fragments were incubated with different concentrations of Fur protein (0–0.05 mM) in EMSA buffer (50 mM Tris-HCl, pH 7.5; 10 mM MgCl_2_; 1 mM DTT; and 100 mM NaCl) at 25 °C for 30 min. Then, the mixed samples were subjected to electrophoresis through an 8% (*w*/*v*) native polyacrylamide gel in 0.5× Tris-borate-EDTA (TBE) buffer (0.044 M Tris base, 0.044 M boric acid, and 0.001 M EDTA, pH 8.0) to separate the protein–DNA complexes from the free DNA. The buffer was pre-run on ice at 80 V for 30 min and then electrophoresed at 150 V for 1 h. After staining with ethidium bromide, visualized bands were obtained through gel imager (BG-gdsAUTO 320) [46,48,52,53]

Further EMSA investigation was conducted on the effects of different concentrations of Fe^2+^ or Fe^3+^ (0, 0.0125, 0.025, and 0.01 mM), and Mn^2+^ (0, 0.125, 0.25, and 0.5 mM) on the binding of Fur protein (0.05 mM) to P*_hemH1_* and P*_hemH2_* promoter DNA fragments (0.001 mM). In addition, the non-transcriptional regulatory protein KatB (catalase) was selected as the negative control for the protein.

To investigate the binding sites of Fur with the P*_hemH1_* and P*_hemH2_* promoters, a DNase I footprinting experiment was conducted. First, fluorescently labeled primers P*_hemH1_*-F (FAM) and P*_hemH2_*-F (FAM) were synthesized (Sangon Biotech, Shanghai, China) (Table S2). We then synthesized the P*_hemH1_* and P*_hemH2_* promoter regions labeled with 5′-FAM by PCR amplification, and incubated the labeled promoter DNA fragments with Fur protein at 25 °C for 30 min to form the protein–DNA complex (described above for EMSA). Then, 5 U of DNase I (Takara, Shanghai, China) was added to the above reaction mixture and incubated at 25 °C for 1 min. Subsequently, 0.25 M EDTA was added to the mixture, and incubated in a 75 °C water bath for 15 min to terminate the reaction. The mixture was then sent to Qingke Sequencing Company for STR sequencing (Beijing, China). According to the sequencing profile analysis, the DNA regions bound by the Fur protein will be protected and cannot be degraded by DNase I, resulting in blank areas forming “footprints”. By comparing these regions with the negative control without Fur protein, the DNA sequence information of the promoters P*_hemH1_* and P*_hemH2_* binding to Fur can be obtained [55,56].

### 4.8. Determination of β-Galactosidase Activity

The PhemH1 and PhemH2 promoter DNA fragments were amplified using primers P*_hemH1_*-F (*Nco* I) and P*_hemH1_*-R (*Bam*H I), and P*_hemH2_*-F (*Nco* I) and P*_hemH2_*-R (*BamH* I), respectively (Table S2). P*_hemH1_* and P*_hemH2_* were then double-digested with *Nco* I and *Bam*H I, respectively, and then ligated into the linearized shuttle plasmid pHT1K-*lacZ* [51,52] to construct the recombinant plasmids pHT1K-*lacZ*-P*_hemH1_* and pHT1K-*lacZ*-P*_hemH2_* (Figure S11). The recombinant plasmids were separately electroporated into Bt 97-27 and Δ*fur* competent cells to obtain four reporter strains for β-galactosidase activity assays: pHP*_h1_*/Bt, pHP*_h1_*/Δ*fur*, pHP*_h2_*/Bt, and pHP*_h2_*/Δ*fur.*

We transferred the four activated reporter strains to 50 mL of LB medium (containing 25 μg/mL Ery) at an initial OD_600_ concentration of 0.01, and cultured them at 28 °C, 200 rpm. When the strains reached the logarithmic growth phase, we took appropriate volumes of samples at intervals to measure the OD_600_, centrifuged them at 8000× *g* for 1 min, and discarded the supernatant. Subsequently, we added 5 mL of Z Buffer to resuspend the cells, sonicated the cell samples on ice, and started timing the reactions after adding 200 μL of ONPG to the treated samples. When a pale yellow reaction appeared, we immediately added 500 μL of Na_2_CO_3_ to stop the reaction and record the reaction time. We measured the absorbance of the sample at 420 and 550 nm, calculated the β-galactosidase activity, and expressed it in Miller units (M.U.) [58].

### 4.9. RNA Extraction and qRT-PCR Analysis

We activated the Bt 97-27 and ∆*hemH2* strains overnight, transferred them at 1%, and incubated them at 28 °C, 200 rpm until the logarithmic growth phase was reached. We used a bacterial RNA extraction kit (Yisheng, Shanghai, China) to extract the total RNA of the Bt 97-27 and ∆*hemH2* strains separately. The final total RNA was analyzed by 1% agarose gel electrophoresis and quantified by NanoDrop (Thermo Scientific, USA). We used Hifair III 1st Strand cDNA Synthesis SuperMix as the qPCR reverse transcription kit (Yeasen, Shanghai, China) to reverse-transcribe RNA into cDNA. Primers were designed using the Primer 5.0 tool and tested for unique binding by Primer-BLAST (Table S3). The gene transcription levels were quantified by real-time qRT-PCR (StepOnePlus Applied Biosystems, Carlsbad, CA, USA) relative to the transcription of reference gene 16S rRNA. We then analyzed the relative expression levels of the genes according to the 2^−ΔΔCt^ method.

### 4.10. Bioinformatics and Statistical Analyses

Amino acid and nucleotide sequences were retrieved from the NCBI database using the BLAST search tool. The ClustalW2 (version 2.1) package (http://www.ebi.ac.uk/Tools/msa/clustalw2/ (accessed on 15 March 2025)) was used for amino acid and nucleotide sequence alignments. All the results data in this study are expressed as mean ± standard error (mean ± SE). Data were plotted and analyzed using GraphPad Prism 8.0 and SPSS 25.0. Statistical differences were firstly determined by one-way ANOVA then by Duncan’s post hoc tests. Among them, “ns” indicates *p* > 0.05, no statistical difference; *p* < 0.05 indicates a statistical difference. “*” indicates *p* < 0.05; “**” indicates *p* < 0.01; “***” indicates *p* < 0.001; “****” indicates *p* < 0.0001.

## 5. Conclusions

Bt 97-27 contains two ferrochelatases, HemH1 and HemH2, which exhibit different function and regulatory mechanisms under various environmental conditions (iron restriction, iron stress, and oxidative stress). Our results demonstrate that HemH2 promotes better growth of recombinant strains under both iron-limited and iron-rich conditions compared with HemH1. HemH1 primarily facilitates heme synthesis under iron-limited conditions by maintaining ALA precursor utilization, while HemH2 drives heme production under iron-replete conditions. Both HemH1 and HemH2 contribute to oxidative stress resistance, with HemH1 conferring greater tolerance to H_2_O_2_ in *E. coli* and Bt 97-27 In terms of bacterial growth, the recombinant strain pEH2/BL grows better than the recombinant strain pEH2/BL under both iron-limited and iron-rich conditions. *hemH1* and *hemH2* both contribute to antioxidant stress in *E. coli* and Bt. EMSA, DNase I footprinting, and β-galactosidase assays all confirmed that Fur can bind to the PhemH1 and PhemH2 promoter DNA, with Fe^2+^ or Fe^3+^ promoting this binding. Fur regulates hemH1 and hemH2 differently under various conditions, suggesting that their expression is jointly regulated by Fur and environmental factors. In summary, HemH1 and HemH2 work synergistically to maintain the physiological homeostasis of Bt 97-27 during bacterial growth, heme biosynthesis, iron concentration stress, and antioxidant stress.

## Data Availability

All relevant data are within the manuscript and available from the authors upon request.

## Supplementary Materials

The following supporting information can be downloaded at: https://www.mdpi.com/article/10.3390/ijms26072911/s1.
