# Supplementary material for: Functional Differentiation and Regulatory Mechanisms of Ferrochelatases HemH1 and HemH2 in Bacillus thuringiensis Under Iron and Oxidative Stress
Source: Int J Mol Sci. 2025 Mar 23;26(7):2911. doi: 10.3390/ijms26072911 (PMC11988928; doi:10.3390/ijms26072911)
Supplement: Supplementary file 1 [file ijms-26-02911-s001.zip › Supplementary materials.pdf]

Supplementary materials

Functional differentiation and regulatory mechanisms of ferrochelatases HemH1 and HemH2 in *Bacillus thuringiensis* under iron and oxidative stress

Jianghan Wang [1,#], Yi Luo [1,#], Tian Jiao [2], Shizhen Liu [1], Ting Liang [1], Huiting Mei [1], Shuang Cheng [1], Qian Yang [1], Jin He [2] and Jianmei Su [1,*]

[1] Hubei Key Laboratory of Regional Development and Environmental Response, Faculty of Resources and Environmental Science, Hubei University, Wuhan 430062, China; 202321108012145@stu.hubu.edu.cn (J.W.); luoyi031622@163.com (Y.L.); 202421108012135@stu.hubu.edu.cn (S.L.); 202231108031008@stu.hubu.edu.cn (T.L.); 202221108012247@stu.hubu.edu.cn (H.M.); 202221108012295@stu.hubu.edu.cn (S.C.); 202131108031001@stu.hubu.edu.cn (Q.Y.)

[2] National Key Laboratory of Agricultural Microbiology, College of Life Science and Technology, Huazhong Agricultural University, Wuhan 430062, China; 2024400382@buct.edu.cn (T.J.); hejin@mail.hzau.edu.cn (J.H.)

* Correspondence: sujianmei@hubu.edu.cn

† These authors contributed equally to this work.

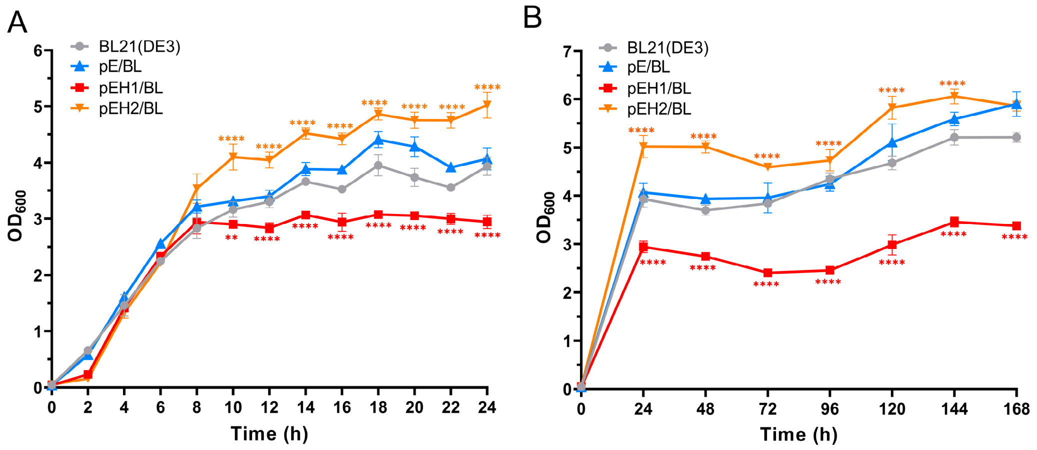

Figure S1. Seven-day growth curves of four *Escherichia coli* strains BL21(DE3), pE/BL, pEH1/BL and pEH2/BL. (A) 24-hour growth curve. (B) seven-day growth curve. Among them, $p < 0.05$ indicates a statistical difference. "*" indicates $p < 0.05$; "**" indicates $p < 0.01$; "***" indicates $p < 0.001$; "****" indicates $p < 0.0001$.

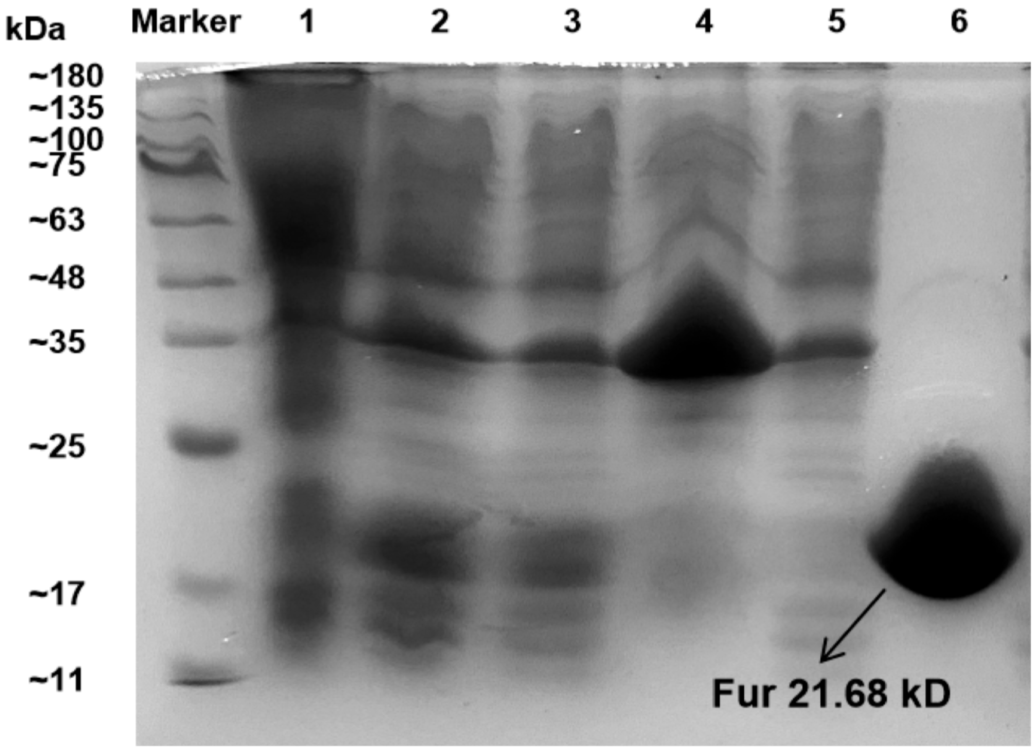

Figure S2. SDS-PAGE gel image of Fur protein. (Marker: Rainbow 180 broad-spectrum protein marker. 1: before induction. 2: after induction. 3: lysate supernatant. 4: lysate pellet. 5: permeate. 6: after dialysis).

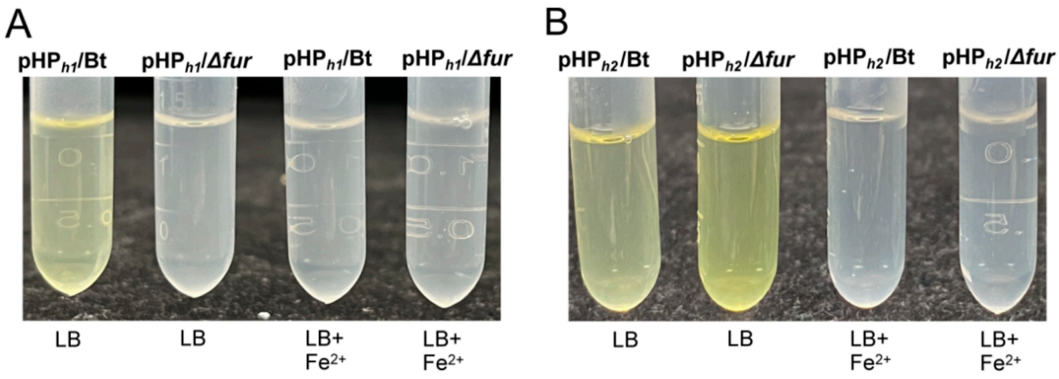

Figure S3. β-galactosidase activity of Bt strains under LB and LB containing Fe^{2+} culture conditions. (A) β-galactosidase activity of pHP$_{h1}$/Bt and pHP$_{h1}$/Δ*fur* strains under 0 and 2.5 mM Fe^{2+} cultivation conditions. (B) β-galactosidase activity of pHP$_{h2}$/Bt and pHP$_{h2}$/Δ*fur* strains under 0 and 2.5 mM Fe^{2+} cultivation conditions.

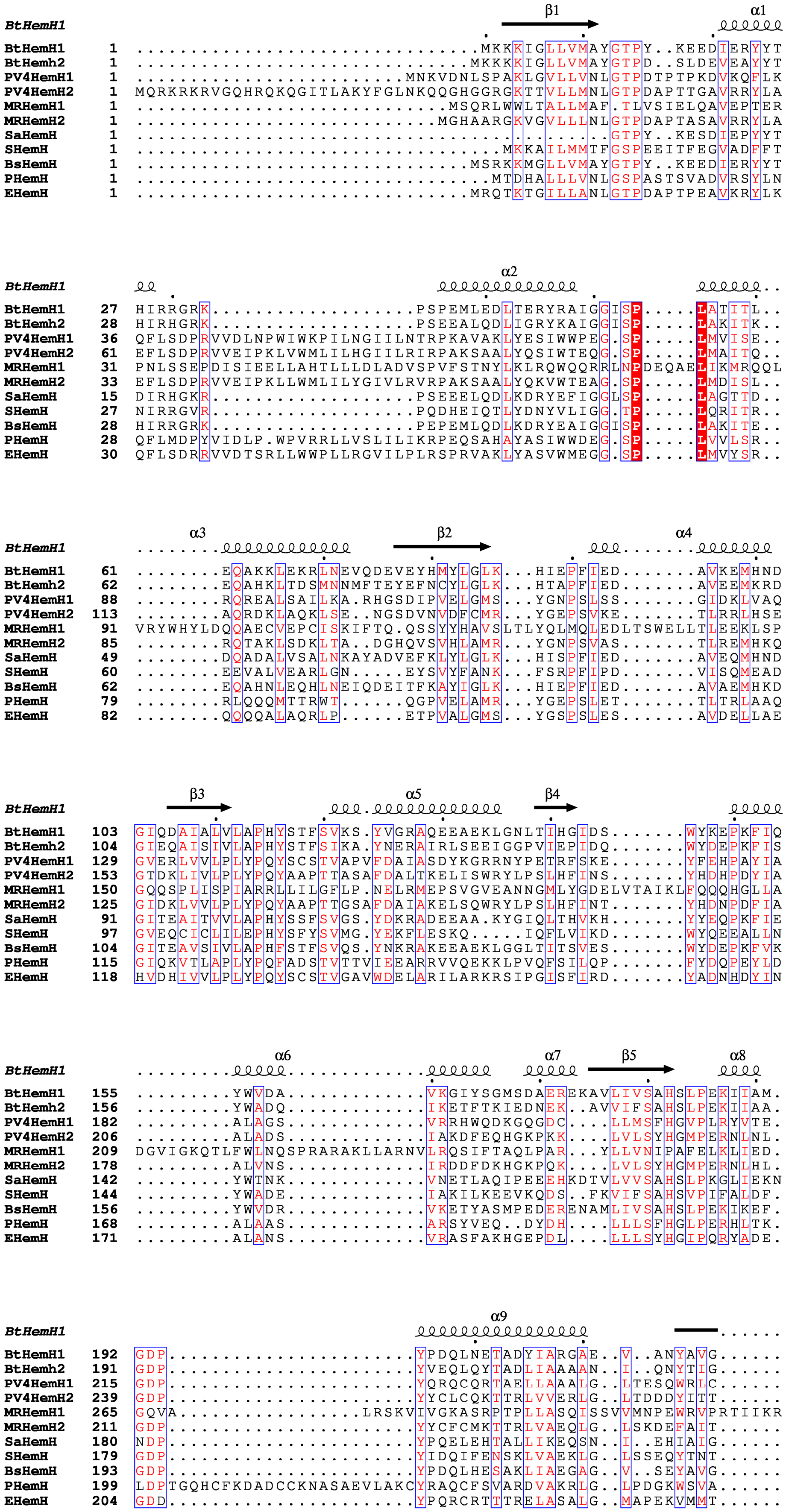

β1
α1
BtHemH1 1 ..............................MKKKIGLLVMAYGTPY..KEEDIERYYT
BtHemh2 1 ..............................MKKKIGLLVMAYGTPD..SLDEVEAYYT
PV4HemH1 1 ........................MNKVDNLSPAKLGVLLVNLGTPDTPTPKDVKQFLK
PV4HemH2 1 MQRKRKRVGQHRQKQGITLAKYFGLNKQQGHGGRGKTTVLLMNLGTPDAPTTGAVRRYLA
MRHemH1 1 ..............................MSQRLWWLTALLMAF.TLVSIELQAVEPTER
MRHemH2 1 ...........................MGHAARGKVGVLLLNLGTPDAPTASAVRRYLA
SaHemH 1 ....GTPY..KESDIEPYYT
SHemH 1 ................................MKKAILMMTFGSPEEITFEGVADFFT
BsHemH 1 ..............................MSRKKMGLLVMAYGTPY..KEEDIERYYT
PHemH 1 ................................MTDHALLLVNLGSPASTSVADVRSYLN
EHemH 1 ..............................MRQTKTGILLANLGTPDAPTPEAVKRYLK
α2
BtHemH1 27 HIRRGRK.................PSPEMLEDLTERYRAIGGISP.....LATITL..
BtHemh2 28 HIRHGRK.................PSEEALQDLIGRYKAIGGISP.....LAKITK..
PV4HemH1 36 QFLSDPRVVDLNPWIWKPILNGIILNTRPKAVAKLYESIWWPEG.SP.....LMVISE..
PV4HemH2 61 EFLSDPRVVEIPKLVWMLILHGIILRIRPAKSAALYQSIWTEQG.SP.....LMAITQ..
MRHemH1 31 PNLSSEPDISIEELLAHTLLLDLADVSPVFSTNYLKLRQWQQRRLNPDEQAELIKMRQQL
MRHemH2 33 EFLSDPRVVEIPKLLWMLILYGIVLRVRPAKSAALYQKVWTEAG.SP.....LMDISL..
SaHemH 15 DIRHGKR.................PSEEELQDLKDRYEFIGGLSP.....LAGTTD..
SHemH 27 NIRRGVR.................PQDHEIQTLYDNYVLIGG.TP.....LQRITR..
BsHemH 28 HIRRGRK.................PEPEMLQDLKDRYEAIGGISP.....LAKITE..
PHemH 28 QFLMDPYVIDLP.WPVRRLLVSLILIKRPEQSAHAYASIWWDEG.SP.....LVVLSR..
EHemH 30 QFLSDRRVVDTSRLLWWPLLRGVILPLRSPRVAKLYASVWMEGG.SP.....LMVYSR..
α3 β2 α4
BtHemH1 61 ........EQAKKLEKRLNEVQDEVEYHMYLGLK...HIEPFIED.......AVKEMHND
BtHemh2 62 ........EQAHKLTDSMNNMFTEYEFNCYLGLK...HTAPFIED.......AVEEMKRD
PV4HemH1 88 ........RQREALSAILKA.RHGSDIPVELGMS...YGNPSLSS.......GIDKLVAQ
PV4HemH2 113 ........AQRDKLAQKLSE..NGSDVNVDFCMR...YGEPSVKE.......TLRRLHSE
MRHemH1 91 VRYWHYLDQQAECVEPCISKIFTQ.QSSYYHAVSLTLYQLMQLEDLTSWELLTLEEKLSP
MRHemH2 85 ........RQTAKLSDKLTA..DGHQVSVHLAMR...YGNPSVAS.......TLREMHKQ
SaHemH 49 ........DQADALVSALNKAYADVEFKLYLGLK...HISPFIED.......AVEQMHND
SHemH 60 ........EEVALVEARLGN.....EYSVYFANK...FSRPFIPD.......VISQMEAD
BsHemH 62 ........EQAHNLEQHLNEIQDEITFKAYIGLK...HIEPFIED.......AVAEMHKD
PHemH 79 ........RLQQQMTTRWT......QGPVELAMR...YGEPSLET.......TLTRLAAQ
EHemH 82 ........QQQQALAQRLP......ETPVALGMS...YGSPSLES.......AVDELLAE
β3 α5 β4
BtHemH1 103 GIQDAIALVLAPHYSTFSVKS.YVGRAQEEAEKLGNLTIHGIDS.......WYKEPKFIQ
BtHemh2 104 GIEQAISIVLAPHYSTFSIKA.YNERAIRLSEEIGGPVIEPIDQ.......WYDEPKFIS
PV4HemH1 129 GVERLVVLPLYPQYSCSTVAPVFDAIASDYKGRRNYPETRFSKE.......YFEHPAYIA
PV4HemH2 153 GTDKLIVLPLYPQYAAPTTASAFDALTKELISWRYLPSLHFINS.......YHDHPDYIA
MRHemH1 150 GQQSPLISPIARRLLILGFLP.NELRMEPSVGVEANNGMLYGDELVTAIKLFQQQHGLLA
MRHemH2 125 GIDKLVVLPLYPQYAAPTTGSAFDAIAKELSQWRYLPSLHFINT.......YHDNPDFIA
SaHemH 91 GITEAITVVLAPHYSSFSVGS.YDKRADEEAA.KYGIQLTHVKH.......YYEQPKFIE
SHemH 97 GVEQCICLILEPHYSFYSVMG.YEKFLESKQ.....IQFLVIKD.......WYQEEALLN
BsHemH 104 GITEAVSIVLAPHFSTFSVQS.YNKRAKEEAEKLGGLTITSVES.......WYDEPKFVK
PHemH 115 GIQKVTLAPLYPQFADSTVTTVIEEARRVVQEKKLPVQFSILQP.......FYDQPEYLD
EHemH 118 HVDHIVVLPLYPQYSCSTVGAVWDELARILARKRSIPGISFIRD.......YADNHDYIN
α6 α7 β5 α8
BtHemH1 155 ........YWVDA.............VKGIYSGMSDAERERKAVLIVSAHSLPEKIIAM.
BtHemh2 156 ........YWADQ.............IKETFTKIEDNEK..AVVIFSAHSLPEKIIAA.
PV4HemH1 182 ........ALAGS.............VRRHWQDKGQGDC....LLMSFHGVPLRYVTE.
PV4HemH2 206 ........ALADS.............IAKDFEQHGKPKK....LVLSYHGMPERNLNL.
MRHemH1 209 DGVIGKQTLFWLNQSPRARAKLLARNVLRQSIFTAQLPAR...YLLVNIPAFELKLIED.
MRHemH2 178 ........ALVNS.............IRDDFDKHGKPQK....LVLSYHGMPERNLHL.
SaHemH 142 ....YWTNK.............VNETLAQIPEEEHKDTVLVVSAHSLPKGLIEKN
SHemH 144 ........YWADE.............IAKILKEEVKQDS..FKVIFSAHSVPIFALDF.
BsHemH 156 ........YWVDR.............VKETYASMPEDERENAMLIVSAHSLPEKIKEF.
PHemH 168 ........ALAAS.............ARSYVEQ..DYDH....LLLSFHGLPERHLTK.
EHemH 171 ........ALANS.............VRASFAKHGEPDL....LLLSYHGIPQRYADE.
α9
BtHemH1 192 GDP.......................YPDQLNETADYIARGAE..V..ANYAVG......
BtHemh2 191 GDP.......................YVEQLQYTADLIAAAAN..I..QNYTIG......
PV4HemH1 215 GDP.......................YQRQCQRTAELLAAALG..LTESQWRLC......
PV4HemH2 239 GDP.......................YYCLCQKTTRLVVERLG..LTDDDYITT......
MRHemH1 265 GQVA...............LRSKVIVGKASRPTPLLASQISSVVMNPEWRVPRTIIKR
MRHemH2 211 GDP.......................YYCFCMKTTRLVAEQLG..LSKDEFAIT......
SaHemH 180 NDP.......................YPQELEHTALLIKEQSN..I..EHIAIG......
SHemH 179 GDP.......................YIDQIFENSKLVAEKLG..LSSEQYTNT......
BsHemH 193 GDP.......................YPDQLHESAKLIAEGAG..V..SEYAVG......
PHemH 199 LDPTGQHCFKDADCCKNASAEVLAKCYRAQCFSVARDVAKRLG..LPDGKWSVA......
EHemH 204 GDD.......................YPQRCRTTTRELASALG..MAPEKVMMT......

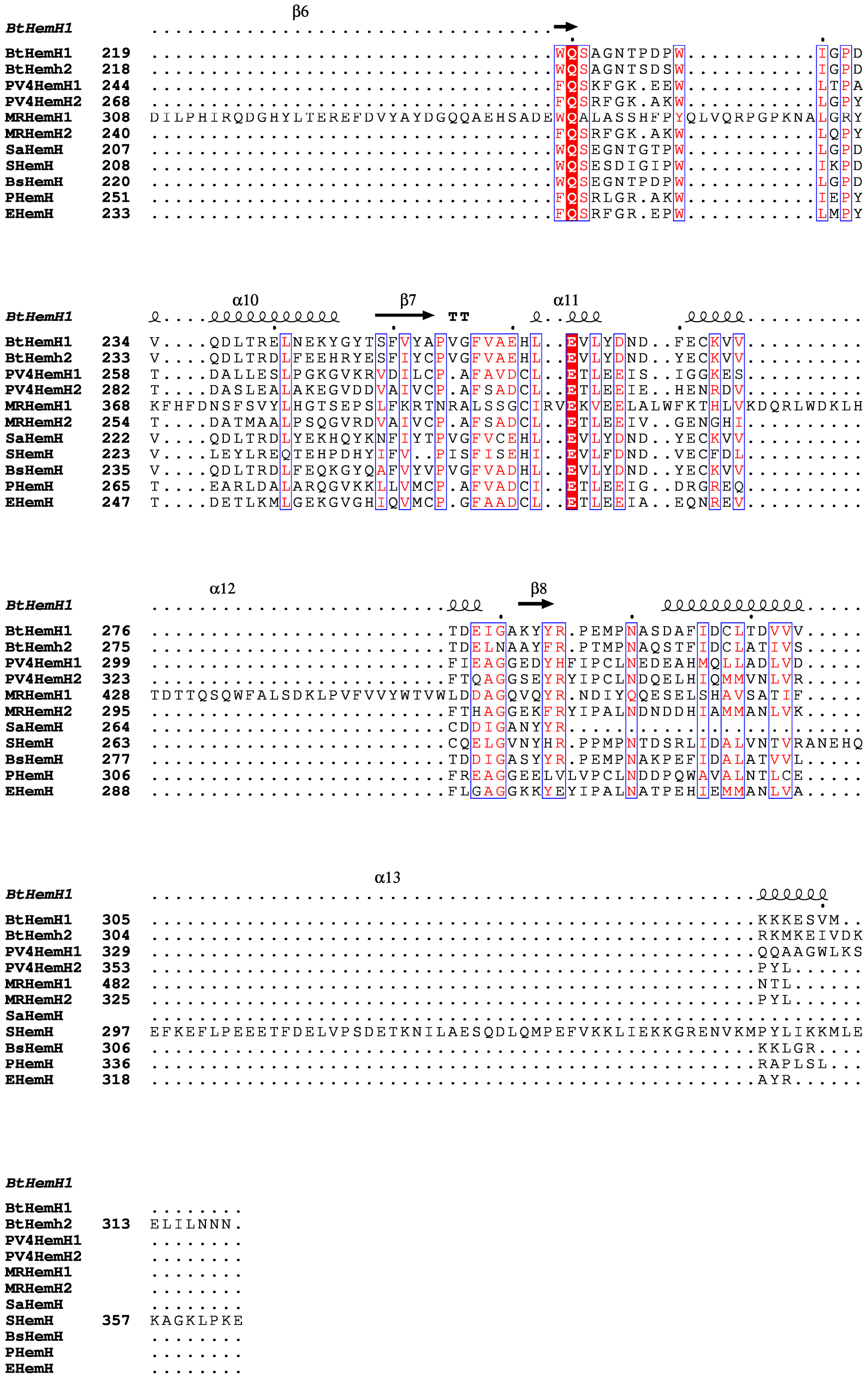

Figure S4. Multiple sequence alignment of the ferrochelatase HemH1 (BtHemH1, AAT62347.1) and HemH2 (BtHemH2, AAT62227.1) from *Bacillus thuringiensis* with ferrochelatases from *Shewanella loihica* PV-4 (PV4HemH1, ABO24095.1) and (PV4HemH2, ABO23011.1) , *Shewanella oneidensis* MR-1 (MRHemH1, AAN55069.2) and (MRHemH2, AAN56346.1), *Staphylococcus aureus* (SaHemH, ABD73483.1), *Shewanella loihica* PV-4 (SaHemH, NC_009092.1), *Streptococcus* (SHemH, WP_023944239.1), *Bacillus subtilis* (BsHemH, SPY20679.1), *Pseudomonas* (PHemH, WP_003171634.1) and *Escherichia coli* BL21(DE3) (EcHemH, CAQ30948.1).

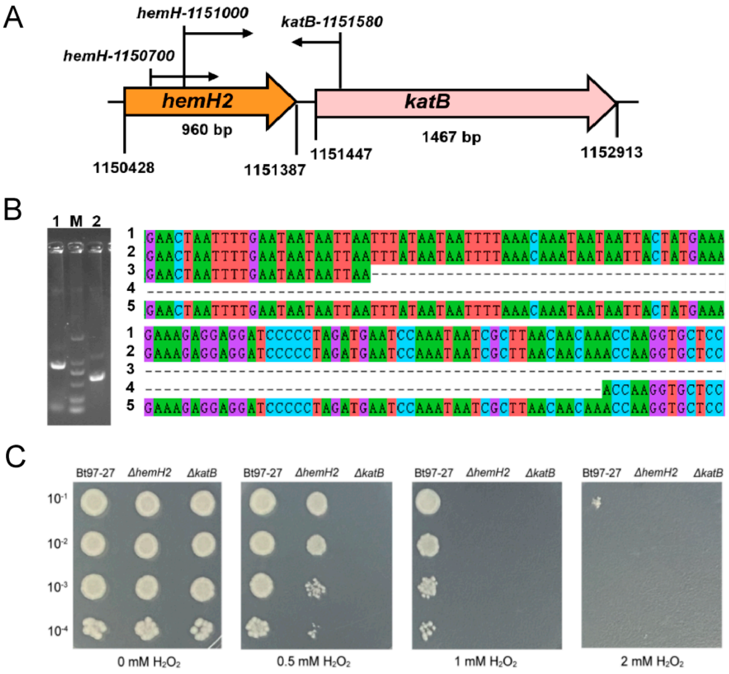

Figure S5. The relationship between *hemH2* gene expression and oxidative stress capacity with the downstream gene *katB*. (A) Schematic diagram of the *hemH2* gene and *katB* gene on the genome and PCR primer design. (B) PCR and sequencing validations of the co-expression of the *hemH2* gene and the *katB* gene using agarose gel electrophoresis. (C) Sensitivity test of Δ*hemH2* and Δ*katB* strains to H_2O_2.

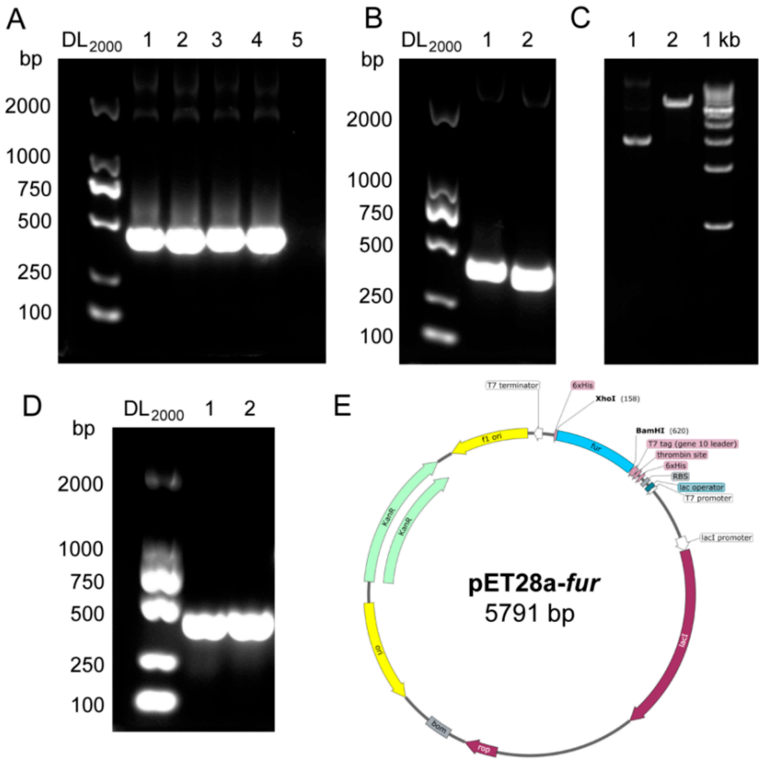

Figure S6. Construction of the pET28a-*fur* recombinant plasmid. (A) Agarose gel electrophoresis of the PCR product of the *fur* gene. (B) Double enzyme digestion of the *fur* gene. Lane 1 is before the double digestion of the *fur* gene, and lane 2 is after the double digestion of the *fur* gene. (C) Double digestion of the pET28a plasmid. Lane 1 is the pET28a before double enzyme digestion, lane 2 is the pET28a after double enzyme digestion (D) PCR verification of the pET28a-*fur* recombinant plasmid by agarose gel electrophoresis. Lanes 1 and 2 are PCR products. (E) pET28a-*fur* recombinant plasmid map. The blue insert is the *fur* gene.

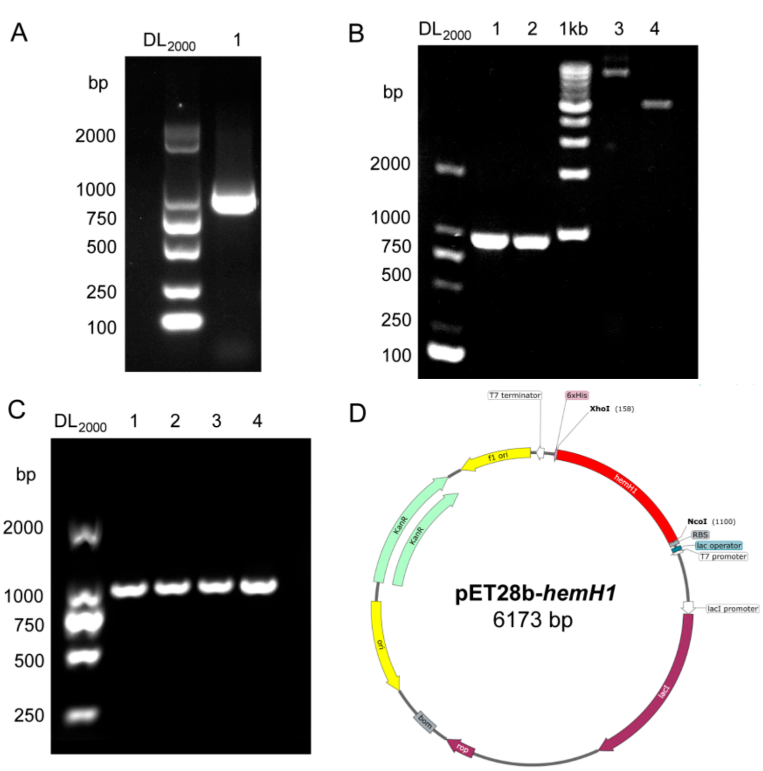

Figure S7. Construction of the pET28b-*hemH1* recombinant plasmid. (A) Agarose gel electrophoresis image of the PCR product of the *hemH1* gene. (B) Double enzyme digestion of the *hemH1* gene and the pET28b plasmid, respectively. Lanes 1 and 2 are the target gene *hemH1* before and after the double enzyme digestion, and lanes 3 and 4 are the vector plasmid pET28b before and after the double enzyme digestion. (C) PCR verification of the recombinant plasmid pET28b-*hemH1* on an agarose gel electrophoresis. Lanes 1-4 are PCR products. (D) pET28b-*hemH1* recombinant plasmid map. The red insert fragment is the *hemH1* gene.

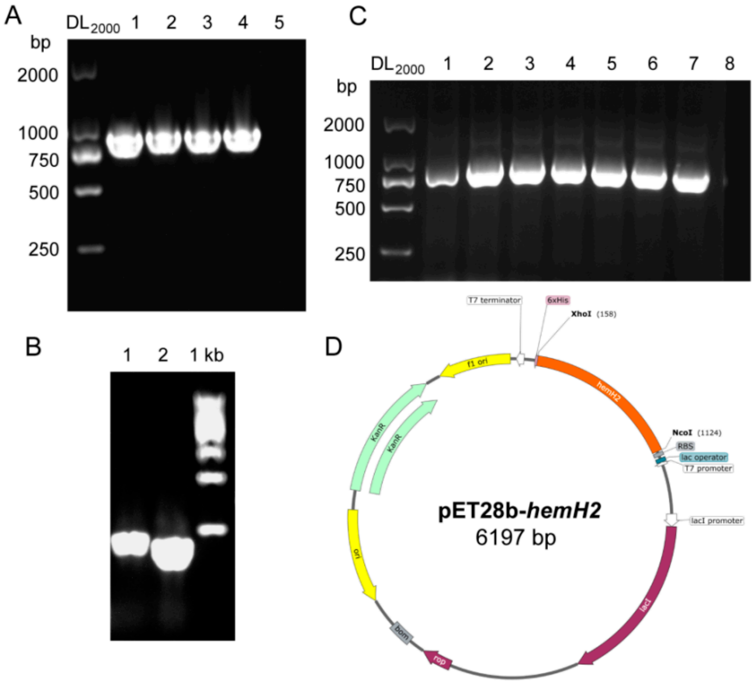

Figure S8. Construction of the pET28b-*hemH2* recombinant plasmid. (A) Agarose gel electrophoresis of the PCR product of the *hemH2* gene. (B) Double enzyme digestion of the *hemH2* gene. lane 1 is before double enzyme digestion of *hemH2*, lane 2 is after double enzyme digestion of *hemH2*. (C) PCR verification of pET28b-*hemH2* recombinant plasmid on agarose gel electrophoresis image, lanes 1-7 are PCR products, lane 8 is the negative control. (D) pET28b-*hemH2* recombinant plasmid map. The orange insert is the *hemH2* gene.

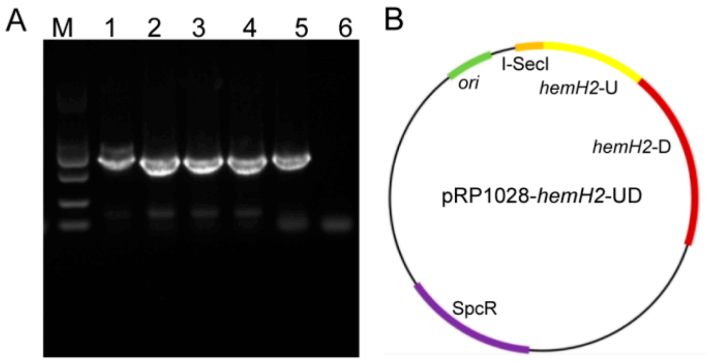

Figure S9. Construction of the *hemH2* gene knockout vector pRP1028-hemH2-UD. (A) Agarose gel electrophoresis image of the PCR product of the *hemH2* gene. M: DL 2000 bp marker, lanes 1-4 are samples of the *hemH2* gene knockout vector, lanes 5-6 are positive and negative controls, respectively. (B) pRP1028-*hemH2*-UD recombinant plasmid map.

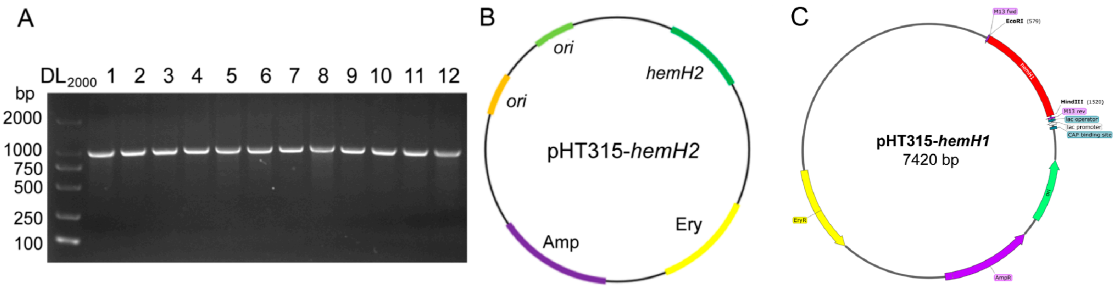

Figure S10. Construction of the pHT315-*hemH2* overexpression vector, plasmid maps of the pHT315-*hemH2* and pHT315-*hemH1* overexpression vectors. (A) Agarose gel electrophoresis image of PCR products from the bacterial culture. Lane 1 and 12 are the positive controls, lanes 2-6 and lanes 7-11 are PCR products from pHT315-*hemH2* and pHT315-*hemH1*, respectively. (B) pHT315-*hemH2* overexpression vector plasmid map. The green insert is the *hemH2* gene. (C) pHT315-*hemH1* overexpression vector plasmid map. The red insert is the *hemH1* gene.

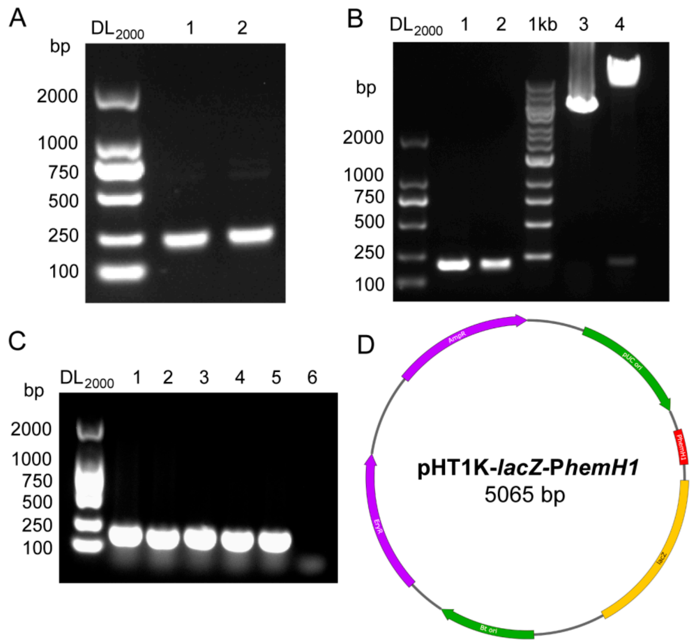

Figure S11. PCR products of the P*hemH1* gene and the recombinant plasmid pHT1K-*lac*Z-P*hemH1*. (A) Agarose gel electrophoresis of the PCR product of the P*hemH1* gene. (B) Double enzyme digestion of the P*hemH1* gene and the pHT1K-*lac*Z plasmid. Lane 1, 2 are the target gene before and after double digestion of P*hemH1*, lanes 3 and 4 are before and after double digestion of the plasmid pHT1K-*lac*Z. (C) PCR verification of the recombinant plasmid pHT1K-*lac*Z-P*hemH1*. Lanes 1-5 are PCR products, and lane 6 is the negative control. (D) Plasmid map of the pHT1K-*lac*Z-P*hemH1* recombinant plasmid. The red insert is the P*hemH1* gene, and the yellow insert is the *lac*Z gene.

Supplementary Tables

Table S1 Primers used in this study

Primer	Sequence
fur-F (*Bam*H I)	CGC*GGATCC*ATGGAAGAAAGAATTGAAC
fur-R (*Xho* I)	GGC*CTCGAG*TTATTTTTCATCCGTTTCAT
hemH1-F (*Nco* I)	CATG*CCATGG*GCATGAAAAAGAAAATTGGTT
hemH1-R (*Xho* I)	CCG*CTCGAG*ACATTACAGATTCTTTTTTCTTTACT
hemH2-F (*Nco* I)	CATG*CCATGG*GCATGAAAAAGAAAAAAATAGGTT
hemH2-R (*Xho* I)	CCG*CTCGAG*ATTATTATTCAAAATTAGTTCT
P_{hemH1}-F (*Nco* I)	CATG*CCATGG*TCTGTTATTGAAG
P_{hemH1}-R (*Bam*H I)	CG*GGATCC*ACAGCAATGCTCCTTTAC
P_{hemH1}-F	TCATTCTTACTTGCGCCATGGTCTG
P_{hemH1}-F (FAM)	TCATTCTTACTTGCGCCATGGTCTG
P_{hemH1}-R	ACAGCAATGCTCCTTTACTTCGC
P_{hemH2}-F (*Nco* I)	CATG*CCATGG*TGGAGTAAATATTAAAGA
P_{hemH2}-R (*Bam*H I)	CG*GGATCC*CTATATAACACCTCAATTTT
P_{hemH2}-F	AGAATAGGAGAAAAGCGACT
P_{hemH2}-F (FAM)	AGAATAGGAGAAAAGCGACT
P_{hemH2}-R	CGTTCCATACGCCATTAC
hemH2-UF (*Kpn* I)	GG*GGTACC*ATTCAAGAAGTTCCTATTTTA
hemH2-UR	ATTATAAACTATATAACACCTCAATTTTATAA
hemH2-DF	TTATATAGTTTATAATAATTTTAAACAAATAA
hemH2-DR (*Mlu* I)	CG*ACGCGT*TGTTTGAATATTTGTATCA
OE*hemH1*-F (*Eco*R I)	CAGTCACGACGTTGTAAAACGACGGCCAGT*GAATTC*CCGCTTGATGTAGTCGGTTTAG
OE*hemH1*-R (*Hin*d III)	ACAGGAAACAGCTATGACCATGATTACGCC*AAGCTT*TTACATTACAGATTCTTTTTTC
OE*hemH2*-F (*Eco*R I)	CAGTCACGACGTTGTAAAACGACGGCCAGT*GAATTC*CCAAACGCCATGGTATGGTC
OE*hemH2*-R (*Hin*d III)	ACAGGAAACAGCTATGACCATGATTACGCC*AAGCTT*TTAATTATTATTCAAAATTAGTTCTTTGTC

katB-1151580	GAATACGTTCACGATCAAAGTGAGCT
hemH-1150700	CATACTGCGCCGTTTATAGAAGATGC
hemH-1151000	TTGCAGCAGGTGACCCTTATGTGGAG

Table S2 Strains and plasmids used in this study

Strain and plasmid	Genotypes or characteristics	Sources
DH5α	*E.coli* DH5α	Invitrogen
pEF/DH	DH5α containing plasmid pET28a-*fur*	This study
pE/DH	DH5α containing plasmid pET28b	This study
pEH1/DH	DH5α containing plasmid pET28b-*hemH1*	This study
pEH2/DH	DH5α containing plasmid pET28b-*hemH2*	This study
pHP$_{h1}$/DH	DH5α containing plasmid pHT1k-P$_{hemH1}$-*lacZ*	This study
pHP$_{h2}$/DH	DH5α containing plasmid pHT1k-P$_{hemH2}$-*lacZ*	This study
pRH2/DH	DH5α containing plasmid pRP1028-*hemH2*-UD	This study
pHH1/DH	DH5α containing plasmid pHT315-*hemH1*	This study
pHH2/DH	DH5α containing plasmid pHT315-*hemH2*	This study
BL21(DE3)	Expressing host *E.coli* BL21(DE3)	Invitrogen
pEF/BL	BL21(DE3) containing plasmid pET28a-*fur*	This study
pE/BL	BL21(DE3) containing plasmid pET28b；Empty vector strain	This study
pEH1/BL	BL21(DE3) containing plasmid pET28b-*hemH1*；*hemH1 recombinant strain*	This study
pEH2/BL	BL21(DE3) containing plasmid pET28b-*hemH2*；*hemH2 recombinant strain*	This study
Bt 97-27	Original strain	This study
Δ*fur*	knockout of *fur* gene in Bt 97-27, *fur* knockout strain	This study
Δ*hemH2*	knockout of *hemH2* gene in Bt 97-27, *hemH2* knockout strain	This study
pH/Bt	Bt 97-27 containing plasmid pHT315；Empty vector strain	This study
pHH2/Bt	Bt 97-27 containing plasmid pHT315-*hemH2*；Overexpression *hemH2*	This study
pHH1/Δ*h2*	Δ*hemH2* containing plasmid pHT315-*hemH1*；*hemH1* complemented strain	This study
pHH2/Δ*h2*	Δ*hemH2* containing plasmid pHT315-*hemH2*；*hemH2* complemented strain	This study
pHP$_{h1}$/Bt	Bt 97-27 containing plasmid pHT1k-P$_{hemH1}$-*lacZ*	This study
pHP$_{h1}$/Δ*fur*	Δ*fur* containing plasmid pHT1k-P$_{hemH1}$-*lacZ*	This study
pHP$_{h2}$/Bt	Bt 97-27 containing plasmid pHT1k-P$_{hemH2}$-*lacZ*	This study
pHP$_{h2}$/Δ*fur*	Δ*fur* containing plasmid pHT1k-P$_{hemH2}$-*lacZ*	This study
pRP1028/DH	DH5α harboring pRP1028	This study
pSS4332/DH	DH5α harboring pSS4332	This study
pSS1827/DH	DH5α harboring pSS1827	This study
plasmid		
pMD19-T	cloning vector,Amp^{+}	Takara
pET28a	expression vector,T7 promoter,Kan^{+}	Qiagen
pET28a-*fur*	pET28a haboring *fur* gene	This study

pET28b	expression vector,T7 promoter, Kan^{+}	Qiagen
pET28b-*hemH1*	pET28b haboring *hemH1* gene	This study
pET28b-*hemH2*	pET28b haboring *hemH2* gene	This study
pHT1k-*lacZ*	pHT1k containing reporter *lacZ* gene,shuttle vector, Amp^{+}, Ery^{+}	This study
pHT1k-P_{hemH1}-*lacZ*	pHT1k-*lacZ* haboring the promoter sequence of *hemH1*	This study
pHT1k-P_{hemH2}-*lacZ*	pHT1k-*lacZ* haboring the promoter sequence of *hemH2*	This study
pRP1028	Spc^{+}	This study
pRP1028-*hemH2*-UD	pRP1028 haboring *hemH2*-UD gene	This study
pHT315	shuttle vector, Amp^{+}, Ery^{+}	Qiagen
pHT315-*hemH1*	pHT315 haboring *hemH1* gene	This study
pHT315-*hemH2*	pHT315 haboring *hemH2* gene	This study
pSS1827	Helper plasmid for conjugative transfer; Amp^{+}	This study
pSS4332	B. *thuringiensis-E. coli* shuttle plasmid; Kan^{+}; containing *gfp* and I-*Sce*I restriction enzyme encoding gene	This study
pRP1028	B. thuringiensis-E. coli shuttle plasmid; Amp^{+}, Ery^{+}, containing temperature-sensitive suicide B. *thuringiensis* replicon, turbo-rfp gene andanI-SceI recognition site, etc	This study

Table S3 Primers used in qRT-PCR

Reference	Primer sequence (F)	Primer sequence (R)
katA	TGTTCACACCAGATCCCTGC	GATCCGGTTTCGAATGTGGC
katB	ACACCAGCAAGCTATCGTGA	ACACCTTGCTGTGGTTTCCA
katB2	GTACGACATGACCAGGGTGG	TGGCCGGAAGAAGAAGTTCC
katX	AAGCGTTGTCACCTTCACCT	ACTATGTCCTGGAATGCGGC
dps	TAAGCCGTTAGCGACGATGA	CCAGCAACTTCCATGCCTTC
ahpC	GCCGATACCGTCAGCATTGA	ACCCAACTCGCACAATCACT
hslO	TGCGTGAGCCGTTTATTGGT	ACAAGGACGCCAACACCTAC
tpx	ACTGCACGAGCAAGTAAACG	TTCGCTCAAAAACGCTGGTG
ohrA	CGGAGAGGCAACAAATCCAG	ATAGGCCGAAACCACCATCTG
BT9727_1785	ACTCAGCAATTGAAGACCGA	AACAAGACAACAACGCGAGC
BT9727_3172	AAGACGCCCCTAATCCCTCT	AGCTGGTTATGGAACCGTACT
BT9727_1760	TCTGCTGAAGGAATTGGGGC	TCTGCTGGTTGTTCGTTTGG
bsaA	CAAGCACCAGGTTTACTCGG	TCTAAATCCACCGGCTTCGT
sodA1	TGGGAAGTAATGAGCCCACG	CCGTCAAGAACAAGCCATCC
sodA2	ACGTTTTTCAGCAGCGTTCC	CAAGCACGCCAAACCAAGAT
sodF	AGGATCAGGCTGGGCAATTC	TACGCATGTTCCCACACGTC
sodC	ATGACACCGCAAGCAATTCG	CGATGCACCGCATATAACGC
gltX	TAGAAGCAGAACGCGAAGGG	ACGCGGAAACGAATACTTGG
hemA	CGTTCAGCACGGTTTGCTTC	AGAAAATGCGCTCTGGTCGT
hemL1（*gsaB*）	GCGATACTTTGTGGTACGCC	TGCTGGTTGTTACCACGGTC
hemL2（*hemL*）	AGCGTTGCTACACCAGAACC	CGCTACGTTTAGCTCGTGGT
hemB	TAATGCGTGGCGAATTGCTG	CGAGTCTCTTGCAGTTCTTGC
hemC	ATCGGAACTTGGCATCCACC	GGCGCATTAGCGATTGAGTG
hemD	CGAAGCGCAACTGGAATGAC	AGCGAGGCTATGAAGTGGAC
hemE	ATTGAAGGCGGTCCATCTCG	TGAACTGCTTTTGCTCCTGC
hemY1	TGTTGTTTTAGCAGCTCCGC	TCCGCCGGTAATTGTTCATCT
hemY2	CGAAATCAGCCATTCAGCGG	GCGTTTTTCCTTCTGGCGTT

hemH1（*cpfC1*）	GCAATGGGCGATCCATATCCA	CGATCCAAGGGTCTGGTGTG
hemH2（*cpfC2*）	GCGATGGAATTGAGCAAGCG	CAGGGCCACCTATTTCCTCC
chdC	CGCCAAGGTGATGACAACTG	ACTTGGCGTACTTTCCCTGC
alaS1	GCGCTCACGTTGTTTTTCCA	TGGAGTAGATGCGTTCCGTC
alaS2	CCACTTCGCAAAGAACCGAC	CCTGTACGTTTCGGGTGTGT
hemN1	TTCACAATTCGGGGCAAACG	AACGATTTTTGTTGGCGGGG
hemN2	CCGGCGTATGCAATTAACGG	CGCCGCCGTAATAAATCGTC
hemX	CGAGAAATGCCCCAATGTTCC	ATCGGGTCTTTTGTCGTTCT
asnB	TCCGTGGATGCGTTCTACAG	TGCTTCTAGCGGACTTTCCC
hmp	TCCGACAACTGAAGCAGAGT	CTGCTGGGCCGAAAAATTCA
isdG	TCAAGTGGAAACAATGCCCG	TTTTGCATTCCAGCGTGTGC
isdE	AATAACTTCCCGGCACACCT	TGGACGTGAGAAACAAGCTG
nos	TGCAGTGGAATGAGAGTCACC	CGACGGAAAAGTGAAACCGAC
gdhA	TAAAGGTGCACGCGTTGTTG	TGGATCATGTAATGCGCCGT
asnA	ACCGAAAGATCGTGAACATGC	ATAATCAGCTGCGCGTCCAT
glnQ	AGGGTCTAAGGCTGAGGTAGG	CTCCCCACCTCCTTTCAGGT
mrp1	TTGGAGAGGGCCTATGCTTG	TCTAGCGCAACATCACCTGT
mrp2	TGCCCATACGAGCTAAAGCC	CGAACATGCTCCGTCCTGAT
glcF	GTCTACGAGGTAAGCTCCGC	GCATTACATGGGCATGCTGG
feoA1	TGCGTAGTGCGATAGTTGTGT	TTAGGATTTATCCCTGGCGCA
feoA2	ACTGTCCACGACACTCCAAT	GAGAAGGCTAGCTGCTTTTGG
feoB1	ACTCCCGCAAACAGTGCATA	GGCAGCGAGAACGATTGAAC
feoB2	TAACAGCACCGATACCACGG	TGCTGAGTTCTTCGGATGGG
feoB3	CCGATGCCTACTCGGTTACG	GATGGAGATGACCAGCAACG
fur	TGCTAATCCGATCTCTGGCG	TTAACACCGCAACGTGAAGC
perR	ACGGAGACGCTTCAAGTAGAT	GTCGTTTTCGCAGCTTCCTC
